# Inhaled AP301 for treatment of pulmonary edema in mechanically ventilated patients with acute respiratory distress syndrome: a phase IIa randomized placebo-controlled trial

**DOI:** 10.1186/s13054-017-1795-x

**Published:** 2017-07-27

**Authors:** Katharina Krenn, Rudolf Lucas, Adrien Croizé, Stefan Boehme, Klaus Ulrich Klein, Robert Hermann, Klaus Markstaller, Roman Ullrich

**Affiliations:** 1 Department of Anaesthesia, Critical Care and Pain Medicine, Medical University of Vienna, Waehringer Guertel 18-20, A-1090 Vienna, Austria; 2 Vascular Biology Center, Department of Pharmacology and Toxicology and Division of Pulmonary and Critical Care Medicine, Medical College of Georgia, Augusta University, Augusta, GA USA; 3 Clinical Research Appliance, Gelnhausen, Germany

**Keywords:** Acute respiratory distress syndrome, Clinical trial, Pulmonary edema, Alveolar fluid clearance, ENaC

## Abstract

**Background:**

High-permeability pulmonary edema is a hallmark of acute respiratory distress syndrome (ARDS) and is frequently accompanied by impaired alveolar fluid clearance (AFC). AP301 enhances AFC by activating epithelial sodium channels (ENaCs) on alveolar epithelial cells, and we investigated its effect on extravascular lung water index (EVLWI) in mechanically ventilated patients with ARDS.

**Methods:**

Forty adult mechanically ventilated patients with ARDS were included in a randomized, double-blind, placebo-controlled trial for proof of concept. Patients were treated with inhaled AP301 (*n* = 20) or placebo (0.9% NaCl; *n* = 20) twice daily for 7 days. EVLWI was measured by thermodilution (PiCCO®), and treatment groups were compared using the nonparametric Mann–Whitney *U* test.

**Results:**

AP301 inhalation was well tolerated. No differences in mean baseline-adjusted change in EVLWI from screening to day 7 were found between the AP301 and placebo group (*p* = 0.196). There was no difference in the PaO_2_/FiO_2_ ratio, ventilation pressures, Murray lung injury score, or 28-day mortality between the treatment groups. An exploratory subgroup analysis according to severity of illness showed reductions in EVLWI (*p* = 0.04) and ventilation pressures (*p* < 0.05) over 7 days in patients with initial sequential organ failure assessment (SOFA) scores ≥11 inhaling AP301 versus placebo, but not in patients with SOFA scores ≤10.

**Conclusions:**

There was no difference in mean baseline-adjusted EVLWI between the AP301 and placebo group. An exploratory post-hoc subgroup analysis indicated reduced EVLWI in patients with SOFA scores ≥11 receiving AP301. These results suggest further confirmation in future clinical trials of inhaled AP301 for treatment of pulmonary edema in patients with ARDS.

**Trial registration:**

The study was prospectively registered at clinicaltrials.gov, NCT01627613. Registered 20 June 2012.

**Electronic supplementary material:**

The online version of this article (doi:10.1186/s13054-017-1795-x) contains supplementary material, which is available to authorized users.

## Background

Acute respiratory distress syndrome (ARDS) is an important cause of respiratory failure in critically ill patients, and it is associated with a high mortality rate of 30% [1, 2]. It frequently progresses to multisystem organ failure [3, 4] and typically evolves within a week following the clinical insult that causes the lung injury [5]. A major characteristic of ARDS is the presence of nonhydrostatic high-permeability pulmonary edema. Impaired alveolar fluid clearance (AFC) in patients with ARDS correlates with mortality and morbidity [6]. However, none of the current therapeutic strategies for ARDS [1, 7–9] is specific or has a direct effect on the molecular mechanisms of fluid clearance from the alveolar space.

AFC strongly depends on the function of amiloride-sensitive epithelial sodium channels (ENaCs) on the apical surface and Na^+^/K^+^-ATPase on the basolateral surface of alveolar epithelial cells [10, 11]. Recently, the cyclic synthetic peptide AP301 (TIP peptide, amino acid sequence: CGQRETPEGAEAKPWYC), which mimics the lectin-like domain of human tumor necrosis factor (TNF)-α (TIP domain), was synthesized, and it was demonstrated to enhance sodium transport by the ENaC [12–15]. The TIP domain does not activate TNF receptors [16, 17] and, in various animal models of lung injury, TIP peptide activated AFC [17–20]. Furthermore, inhaled TIP peptide reduced the extravascular lung water index (EVLWI) in acute lung injury in pigs [21, 22]. ENaC activity depends on the product of the surface expression *N* and the open probability *Po*. TIP peptide increases both *N* and *Po* in the absence or presence of the bacterial toxin pneumolysin, a major virulence factor from *Streptococcus pneumoniae* [23]. Apart from activating AFC, the TIP peptide was also shown to strengthen barrier function in human lung microvascular endothelial monolayers [20].

A first-in-man study demonstrated the excellent tolerability of inhaled AP301 and minimal systemic absorption of the peptide [24]. To assess the clinical effect of inhaled AP301 on EVLWI, we performed a randomized, placebo-controlled, proof-of-concept pilot trial in 40 mechanically ventilated patients with ARDS.

## Methods

### Participants and setting

The study (Ethics Committee No. 1424/2012) was approved by the ethics committee of the Medical University of Vienna, Vienna, Austria (Chairperson: Prof. E. Singer) on 12 June 2012 and was prospectively registered at clinicaltrials.gov (NCT01627613). Informed consent was obtained from all study participants according to the Austrian legislation that regulates the consent of nonconscious subjects included in clinical trials. National legalization requires patients to provide informed consent immediately after they regain consciousness. In case that a court-appointed legal representative has been determined, this formal legal representative needs to consent prior to randomization.

We included patients (age ≥18 years) with ARDS within 48 h of diagnosis who required intubation and mechanical ventilation. The study was performed from August 2012 to February 2014 at seven surgical and anesthesiological intensive care units (ICUs) of the Medical University of Vienna covering a total of 58 beds that predominantly serve surgical or trauma patients. Patients who met the criteria of the European-American consensus conference (bilateral pulmonary infiltrates on frontal chest x-ray, PaO_2_/FiO_2_ ratio ≤300 mmHg, and a pulmonary capillary wedge pressure ≤18 mmHg or no clinical evidence of left atrial hypertension) [25], had an EVLWI ≥8 mL/kg predicted body weight (PBW), had a negative pregnancy test (for females of child-bearing potential), and presented with stable hemodynamics for at least 8 h were included. The exclusion criteria were as follows: brainstem death, cardiogenic pulmonary edema, current evidence of septic shock as defined by the Surviving Sepsis Campaign criteria, neutrophil count <0.3 × 10^9^/L, immunosuppression (i.e., high-dose steroids: prednisolone >80 mg/day or hydrocortisone >300 mg/day, cancer treatment including chemotherapy or biological or immunosuppressive therapy for organ transplantation within 2 weeks), body mass index <18.5 or >35 kg/m^2^, active pregnancy, and participation in other interventional trials. Patients received randomized study treatment on top of standard of care.

### Study design

The present study was a single-center, randomized, double-blind, placebo-controlled clinical trial (*n* = 20 AP301 inhalation, *n* = 20 placebo 0.9% saline inhalation). The study protocol (Additional file 1) defined stratification according to severity of illness by sequential organ failure assessment (SOFA) score [26] at screening with allocation of patients with SOFA scores ≤10 to stratum A and patients with SOFA scores ≥11 to stratum B. Randomization was performed using separate randomization lists for strata A and B that were prepared by Bioconsult GmbH (Breitenfurt, Austria) and known only to the local pharmacy at the Medical University of Vienna where enrolled patients were assigned to treatment groups, and blinded study drugs were prepared. The randomization method for both strata was block randomization using random computer-generated permuted blocks with block sizes of one to three patients. KK, RU, Petra Erlinger, and Thomas Kollarits performed enrollment and data collection. Inhalations (AP301 or 0.9% saline) were started in the evening of the day of screening or the next morning if randomization was performed after 12 am. Patients received study treatment every 12 ± 0.5 h for 7 days (total of 14 doses). Inhalation was stopped prematurely in cases of extubation or if treatment had to be discontinued for clinical reasons, including the occurrence of serious adverse events (SAEs) or appearance of exclusion criteria. Blinded study drug preparations consisted of 5 mL clear liquid solution containing either 125 mg AP301 (Apeptico GmbH, Vienna, Austria) dissolved in water or 0.9% NaCl, both of which were injected into the nebulizer chamber of an Aeroneb solo device (Aerogen, Galway, Ireland) connected to the inspiratory limb of the breathing circuit. The nebulizer filling dose of 125 mg AP301 was less than the highest dose applied in the phase I trial [24].

### Data collection and measurements

The primary efficacy variable EVLWI was measured using transpulmonary thermodilution (PiCCO®, Pulsion Medical Systems, Munich, Germany) 1–2 h after each inhalation. This was performed via the injection of 20 mL 0.9% saline at 4 °C through the central venous catheter and temperature measurement using a femoral arterial thermistor catheter. The arithmetic mean of three consecutive EVLWI measurements was used as the respective time point value. The “last observation carried forward” (LOCF) method was used to impute missing values [27–29] in patients with at least seven actually measured EVLWI values. Ventilation parameters, blood gas analysis, and Murray lung injury score (LIS) [30] were recorded once daily until day 7. At screening, the gas exchange, organ failure, cause, and associated conditions (GOCA) score was recorded [31]. Length of mechanical ventilation, adverse events (AEs), and survival were documented until day 28. Safety was assessed via daily laboratory analyses (white blood cell count, hemoglobin, hematocrit, platelet count, creatinine, sodium, potassium, blood urea nitrogen, and bilirubin) and assessments of vital signs (pulse rate, oxygen saturation, blood pressure, and cardiac index measured by PiCCO®) until day 7.

### Outcomes

The primary outcome parameter was the mean baseline-adjusted EVLWI change from screening to day 7 of treatment. The mean baseline-adjusted EVLWI change was calculated for each patient as the arithmetic mean of 15 differences between EVLWI at screening (*t* 0) and that at each respective time point (*t* 0 to *t* 14). Secondary outcome parameters were the correspondingly calculated mean differences in baseline adjusted PaO_2_/FiO_2_ ratio, peak ventilation pressure, ventilatory plateau pressure, mean airway pressure, positive end-expiratory pressure (PEEP), and Murray LIS from screening to day 7. Ventilator-free days were calculated as the number of days for which ventilator support was not provided until day 28. Patients with tracheostomy with pressure support and PEEP ≤8 cmH_2_O were considered free from ventilator support [32]. In addition, an exploratory subgroup analysis of the SOFA score strata was performed to compare primary and secondary outcome parameters between the treatment groups within each stratum.

### Statistics

A mean difference in EVLWI (baseline SD = 40%) of at least 40% was considered to indicate a clinically relevant effect of AP301 versus placebo. Power analysis yielded a sample size of 40 (20/group) with *p* < 0.05 (two-sided) and a power of 80%. The primary efficacy variable (mean baseline-adjusted difference in EVLWI from screening to day 7) was calculated as described in the section “Outcomes” and analyzed using the nonparametric Mann–Whitney *U* test. Secondary efficacy parameters were analyzed using nonparametric tests appropriate for the type and distribution of data (Mann–Whitney *U* test and Pearson chi-square (χ^2^) test). Continuous parameters are presented as the mean ± SD, ventilator-free days and length of ICU stay are presented as the median with interquartile range (IQR), and categorical data are reported as percentages. All statistical analyses were performed using the SPSS software.

## Results

### Patient randomization and demographics

**Fig. 1:**
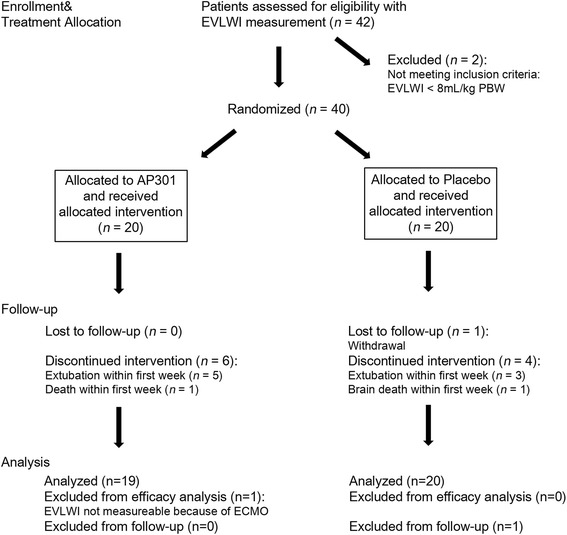
Consort flow chart showing study progress from enrollment to analysis. *EVLWI* extravascular lung water index, *PBW* predicted body weight

Mechanically ventilated ICU patients were screened for eligibility during the study period from August 2012 to February 2014. Forty-two patients were screened via PiCCO® measurements, and 40 of these patients were randomized after fulfilling all inclusion criteria and missing all exclusion criteria (Fig. 1). Randomization occurred within 48 h of ARDS diagnosis in all included patients. Eight patients randomized to the AP301 group and seven randomized to the placebo group had a SOFA score ≤10 at screening (stratum A). Twelve patients randomized to AP301 and 13 patients randomized to placebo had a SOFA score ≥11 at screening (stratum B). Twelve of 14 study treatment inhalations were completed in both treatment arms. Of the 14 scheduled EVLWI measurements, 12 were performed in the AP301 group versus 13 in the placebo group. The most reported reason for missing EVLWI values that were imputed using the LOCF method was removal of the PiCCO catheter on day 6 or 7 of the study, which occurred mostly in extubated patients. The second most reported reason was technical problems with the measurement, especially in patients on extracorporeal membrane oxygenation (ECMO) therapy. Missing values were not substituted in two patients who died within 7 days and in one patient with less than seven available EVLWI measurements due to extubation and removal of the PiCCO catheter. One patient had to be excluded from the efficacy analysis owing to repeated technical interferences of ECMO with thermodilution. All other 39 patients were analyzed according to the intention-to-treat principle. One patient refused to complete follow-up, requiring exclusion at day 8. Eight patients were extubated before completing day 7 of therapy (five in the AP301 and three in the placebo group). One patient in each treatment group died within the first week. During the ensuing observation period from day 8 to day 28, eight additional patients died (five and three in the AP301 and placebo groups, respectively).

Baseline demographic parameters and GOCA scores of patients are shown in Table 1. Seventy-five percent of patients in both treatment groups had surgical diagnoses, and 25% had medical diagnoses. Ventilation parameters and blood gas analysis results at screening were equally distributed between treatment groups (Table 2) with the exception of four patients in the AP301 group who had received ECMO therapy at screening versus one patient in the placebo group. All patients who had received ECMO therapy at screening were included in stratum B (SOFA score ≥11). Overall, patients with SOFA score ≥11 had slightly lower PaO_2_/FiO_2_ ratios as well as higher Murray LIS and oxygenation indices in both treatment groups (Table 3) than those with SOFA score ≤10. The causes of ARDS in study participants are shown in Table 4.

**Table 1:** Patient characteristics at screening

Parameter	AP301 (*n* = 20)	Placebo (*n* = 20)
Age, years	47.6 ± 17.4	50.2 ± 14.9
Gender, male/female	14 (70)/6 (30)	12 (60)/8 (40)
BMI, kg/m^2^	26.1 ± 3.7	28.5 ± 5.2
SOFA score	12.5 ± 3.6	11.7 ± 3.4
Surgical	15 (75)	15 (75)
Medical	5 (25)	5 (75)
GOCA		
Gas exchange		
PaO_2_/FiO_2_ ratio*		
201–300 mmHg (mild)	2 (10)	3 (15)
101–200 mmHg (moderate)	14 (70)	11 (55)
≤ 100 mmHg (severe)	4 (20)	6 (30)
PEEP		
0–5 cmH_2_O	0 (0)	0 (0)
6–10 cmH_2_O	9 (45)	11 (55)
>10 cmH_2_O	11 (55)	9 (45)
Organ failure		
Lung only	11 (55)	10 (50)
Lung + 1 organ	4 (20)	7 (35)
Lung + 2 organs	4 (20)	3 (15)
Lung + 3 organs	1 (5)	0 (0)
Cause		
Direct	12 (60)	12 (60)
Indirect	8 (40)	8 (40)
Associated diseases		
No coexisting diseases	12 (60)	16 (80)
Coexisting diseases causing death within 5 years	6 (30)	4 (20)
Coexisting diseases causing death within 6 months	2 (10)	0 (0)

Data are presented as mean ± SD or *n* (%)

*Identical PaO_2_/FiO_2_ cutoff values are used in the Berlin Definition of ARDS severity [5]

*BMI* body mass index, *GOCA* gas exchange, organ failure, cause, and associated diseases, *PEEP* positive end-expiratory pressure, *SOFA* sequential organ failure assessment

**Table 2:** Respiratory parameters at screening

Parameter	AP301 (*n* = 20)	Placebo (*n* = 20)
PaO_2_/FiO_2_, mmHg	147 ± 48	150 ± 59
Murray LIS	2.9 ± 0.5	2.8 ± 0.5
Oxygenation index	12.8 ± 5.5	10.6 ± 5.0
Prone positioning	6 (30)	5 (25)
ECMO therapy	4 (20)	1 (5)
EVLWI, mL/kg PBW	13.6 ± 5.6	12.5 ± 5.0
Peak ventilator pressure, cmH_2_O	26 ± 4	25 ± 4
Driving pressure, cmH_2_O	14 ± 3	14 ± 4
Mean airway pressure, cmH_2_O	17 ± 4	15 ± 3
PEEP, cmH_2_O	12 ± 3	11 ± 2
V_T_, mL/kg PBW	6.7 ± 1.7	7.8 ± 1.6
Respiratory rate, per min	18 ± 6	18 ± 3
PaCO_2_, mmHg	46 ± 7	44 ± 9
pH	7.37 ± 0.10	7.40 ± 0.07

Data are presented as mean ± SD or *n* (%)

*ECMO* extracorporeal membrane oxygenation, *EVLWI* extravascular lung water index, *LIS* lung injury score, *PBW* predicted body weight, *PEEP* positive end expiratory pressure, *V*_*T*_ tidal volume

**Table 3:** Respiratory parameters at screening according to SOFA stratification

	SOFA A		SOFA B	
Parameter	AP301 (*n* = 8)	Placebo (*n* = 7)	AP301 (*n* = 12)	Placebo (*n* = 13)
PaO_2_/FiO_2_, mmHg	180 ± 63	188 ± 46	147 ± 55	156 ± 44
Murray LIS	2.5 ± 0.4	2.6 ± 0.3	3.2 ± 0.3	2.9 ± 0.6
Oxygenation index	9.3 ± 4.5	7.8 ± 2.2	15.1 ± 5.1	12.1 ± 5.5
Prone positioning	2 (25)	0	4 (33.3)	5 (38.5)
ECMO therapy	0	0	4 (33.3)	1 (7.8)
EVLWI, mL/kg PBW	10.2 ± 1.4	12.7 ± 4.3	15.8 ± 6.2	12.5 ± 5.5
Peak ventilator pressure, cmH_2_O	23 ± 3	23 ± 5	28 ± 4	26 ± 4
Driving pressure, cmH_2_O	14 ± 3	13 ± 5	15 ± 3	15 ± 4
Mean airway pressure, cmH_2_O	14 ± 2	14 ± 1	19 ± 4	16 ± 3
PEEP, cmH_2_O	10 ± 2	10 ± 1	13 ± 3	11 ± 2
V_T_, mL/kg PBW	7.7 ± 1.3	8.0 ± 1.3	6.0 ± 1.5	7.6 ± 1.7
Respiratory rate, per min	16 ± 2	17 ± 2	19 ± 7	18 ± 4
PaCO_2_, mmHg	45 ± 5	40 ± 3	48 ± 7	47 ± 10
pH	7.43 ± 0.07	7.43 ± 0.03	7.33 ± 0.10	7.38 ± 0.08

Data are presented as mean ± SD or *n* (%)

*ECMO* extracorporeal membrane oxygenation, *EVLWI* extravascular lung water index, *LIS* lung injury score, *PBW* predicted body weight, *PEEP* positive end expiratory pressure, *SOFA* sequential organ failure assessment, *V*_*T*_ tidal volume

**Table 4:** Causes of acute respiratory distress syndrome

Condition	Assignment to groups (*n*)	*n* (%)
Multiple trauma	AP301: 2, Placebo: 3	5 (12.5)
Pneumonia	AP301: 3, Placebo: 2	5 (12.5)
Sepsis	AP301: 1, Placebo: 3	4 (10)
Subarachnoidal hemorrhage	AP301: 1, Placebo: 2	3 (7.5)
Respiratory failure following abdominal surgery	AP301: 3	3 (7.5)
Burn injury >40% of body surface	AP301: 3	3 (7.5)
Perforation of the small intestine or colon	AP301: 2, Placebo: 1	3 (7.5)
Traumatic brain injury	Placebo: 2	2 (5)
Cerebral ischemia	AP301: 1, Placebo: 1	2 (5)
Other causes	AP301: 4, Placebo: 6	10 (25)

### Primary efficacy variable

**Fig. 2:**
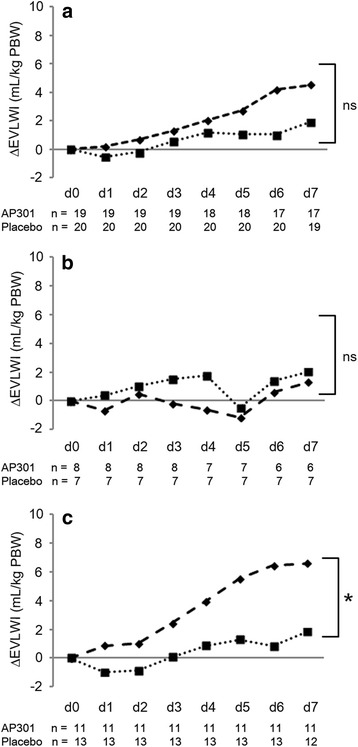
Baseline-adjusted differences in extravascular lung water index (*EVLWI*) over 7 days. Comparison of 7-day (*d0–d7*) treatment with inhaled AP301 (*dashed line*) or placebo (*dotted line*). Data are given as mean and patient count per day. A positive ΔEVLWI indicates a decrease from baseline value at screening. **a** AP301 versus placebo. **b** AP301 versus placebo in stratum A (SOFA score ≤10). **c** AP301 versus placebo in stratum B (SOFA score ≥11). *p = 0.04. *ns* not significant, *PBW* predicted body weight

The mean baseline-adjusted EVLWI reduction from screening to day 7 was 2.0 ± 4.2 mL/kg PBW in the AP301 group, compared with 0.7 ± 2.9 mL/kg PBW in the placebo group (*p* = 0.196). The course of mean daily differences between EVLWI at screening and each study day is shown in Fig. 2 and summarized in Table 5.

**Table 5:** Mean daily differences between extravascular lung water index (EVLWI) at screening and respective study day

Group	Day 1	Day 2	Day 3	Day 4	Day 5	Day 6	Day 7
AP301	0.2 ± 3.1	0.7 ± 3.7	1.3 ± 4.5	2.0 ± 6.3	2.7 ± 6.7	4.2 ± 5.9	4.5 ± 5.6
Placebo	–0.5 ± 3.3	–0.2 ± 3.3	0.6 ± 3.1	1.2 ± 3.6	1.0 ± 4.7	1.0 ± 6.1	1.9 ± 4.1
Stratum A AP301	–0.7 ± 4.3	0.5 ± 3.7	–0.2 ± 3.4	–0.6 ± 7.4	–1.2 ± 7.2	0.6 ± 3.9	1.3 ± 2.6
Stratum A placebo	0.4 ± 0.5	1.0 ± 1.6	1.5 ± 2.4	1.8 ± 1.9	–0.5 ± 8.8	1.4 ± 4.5	2.0 ± 3.8
Stratum B AP301	0.9 ± 1.7	1.0 ± 3.9	2.4 ± 5.0	4.0 ± 4.9	5.5 ± 4.9	6.4 ± 6.0	6.6 ± 6.1
Stratum B placebo	–1.0 ± 4.1	–0.9 ± 3.8	0.1 ± 3.5	0.9 ± 4.2	1.3 ± 4.3	0.8 ± 7.0	1.8 ± 4.5

EVLWI data are expressed as mL/kg predicted body weight and presented as mean ± SD

### Secondary outcome parameters

There were no significant differences in PaO_2_/FiO_2_ ratios, ventilation pressures, or Murray LIS between the treatment groups (Table 6). In an exploratory analysis of secondary outcomes, 15 (IQR 9–21) ventilator-free days were recorded for patients treated with AP301, versus 12 (IQR 0–20) in the placebo group (*p* = 0.22). The duration of ICU stay did not differ between the treatment groups (24.5 (IQR 15–28) days in the AP301 group versus 24.0 (IQR 16–28) days in the placebo group).

**Table 6:** Clinical parameters at screening and after 7 days of treatment with inhaled AP301 or placebo

	Screening		Day 7	
Parameter	AP301	Placebo	AP301	Placebo
HR, per min	90 ± 18	83 ± 22	96 ± 23	84 ± 20
SBP, mmHg	112 ± 12	121 ± 20	121 ± 14	133 ± 25
CI, L/min/m	3.8 ± 1.2	3.3 ± 0.9	3.9 ± 1.2	3.7 ± 0.9
Noradrenalin dose, mg/24 h	16.3 (0–86.3)	9.5 (0–72.1)	5.6 (0–32.0)	3.0 (0–25.6)
PBV, mL	351 ± 109	384 ± 180	322 ± 89	406 ± 228
EVLWI, mL/kg PBW	13.6 ± 5.6	12.5 ± 5.0	9.0 ± 2.2	11.5 ± 5.1
24-h fluid balance, mL	1307 ± 1525	975 ± 1732	–421 ± 1183	–467 ± 1102
Peak ventilator pressure, cmH_2_O	26 ± 4	25 ± 4	21 ± 7	21 ± 7
Driving pressure, cmH_2_O	14 ± 3	14 ± 4	9 ± 5	10 ± 4
PEEP, cmH_2_O	12 ± 3	11 ± 2	10 ± 4	10 ± 2
PaO_2_/FiO_2_, mmHg	147 ± 48	150 ± 59	211 ± 60	192 ± 60
Murray LIS	2.9 ± 0.5	2.8 ± 0.5	2.0 ± 0.7	2.1 ± 0.6
Prone positioning	6 (30)	5 (25)	2 (10)	3 (15)
ECMO therapy	4 (20)	1 (5)	0	1 (5)

Data are presented as mean ± SD, mean (absolute range), or *n* (%)

*CI* cardiac index, *ECMO* extracorporeal membrane oxygenation, *EVLWI* extravascular lung water index, *HR* heart rate, *LIS* lung injury score, *PBV* pulmonary blood volume, *PBW* predicted body weight, *PEEP* positive end-expiratory pressure, *SBP* systolic blood pressure

We observed an overall mortality of 25% during the study period of 28 days (10 deaths out of 40 study patients). There were six deaths in the AP301 group (30%, three each in stratum A and B) and four deaths in the placebo group (20%, all in stratum B).

### Additional analyses

In an exploratory subgroup analysis of SOFA score strata, there were no differences in EVLWI or secondary outcomes between treatment groups in stratum A. Interestingly, in stratum B, the mean baseline-adjusted change in EVLWI between screening and day 7 was higher in patients treated with AP301 (3.6 ± 3.7 mL/kg PBW) than in those treated with placebo (0.4 ± 3.4 mL/kg PBW; *p* = 0.04). Furthermore, we observed a reduction in peak ventilation pressure (*p* = 0.018), plateau pressure (*p* = 0.01), PEEP (*p* = 0.022), and mean airway pressure (*p* = 0.01) in patients treated with AP301 versus placebo in stratum B. Patients inhaling AP301 in stratum B had 21 (IQR 15–21) ventilator-free days versus 11 (IQR 0–17) days in the placebo group (*p* = 0.06). This trend started within the first week. Four patients were extubated between days 4 and 7 in the AP301 group whereas one patient was extubated on day 7 in the placebo group.

### Adverse events

The tolerability and safety of the treatment were generally good. There were no differences in the total numbers of AEs and SAEs between the treatment groups. A decreased tidal volume on pressure-controlled ventilation occurring immediately after inhalation of AP301 in a 67-year-old male patient with 60% burn injury was the only possible treatment-associated AE that could be identified. After treatment with inhaled bronchodilators and steroids as well as bronchoscopy, the patient was weaned to an assisted ventilation mode on the same day, and he did not exhibit any adverse reactions to subsequent AP301 treatment. The most frequent AEs within 28 days were tracheostomy, anemia, and worsening of preexisting anemia (Table 7). Taken together, new onset of anemia and worsening of anemia that was present at screening occurred equally in eight patients per treatment group. Thrombopenia in three patients in the AP301 group was most likely related to liver dysfunction, as two of three patients also had increased bilirubin levels at screening. Leukocytosis occurred in three patients in the AP301 group versus one patient in the placebo group, who presented with concomitant thrombopenia. Possible explanations for leukocytosis in the AP301 group are earlier splenectomy and recent surgery, aspiration pneumonia, and pneumonia in a patient with burn injuries. The hematological abnormalities observed in the AP301 group are commonly seen during the postoperative period, during infection or liver dysfunction. However, an association with AP301 therapy cannot be excluded at this stage. Cardiac arrest occurred in five patients in the AP301 group. One cardiac arrest occurred within the active treatment period of 7 days in a patient with 60% burn injury and inhalation trauma who developed severe hypoxia and multiorgan failure. This patient received renal replacement and ECMO therapy and required increasing doses of catecholamines because of hemodynamic instability. Study inhalations were stopped 2 days before cardiac arrest because the unstable condition prohibited any preventable manipulation of the breathing circuit. In addition, one patient in the placebo group died within the active treatment period. This patient was pronounced brain dead after subarachnoidal hemorrhage complicated by increasing intracranial pressure. This patient was excluded from the study on day 6 because the exclusion criterion of “brainstem death” was observed. The other four fatal cardiac arrests in the AP301 group occurred after day 7. They were not related to study therapy but caused by the severity of the underlying critical illness. In three of these patients, cardiac arrest followed the decision to limit further intensive care treatment owing to futility, i.e., no cardiopulmonary resuscitation (CPR) in two patients (on days 15 and 18 after screening) and additional withdrawal of renal replacement therapy in one patient (on day 13 after screening). Their underlying conditions were 60% burn injury, treatment refractory epileptic status, and acute kidney failure together with thrombotic and bleeding complications after resection of a large thoracic tumor, respectively. Unsuccessful CPR was performed in one patient with aspiration pneumonia as a cause of ARDS who developed acute myocardial ischemia after emergency surgery on day 11 after screening and had a history of coronary artery disease. One cardiac arrest with successful CPR occurred in the placebo group on day 1 of study treatment. The only differences in laboratory parameters or vital signs were higher bilirubin levels at screening in the AP301 group, as this group included more patients with liver dysfunction. The cumulative dose of furosemide within 7 days was higher in the AP301 group in stratum A (477 ± 284 mg, range 40–804 mg, versus 120 ± 85 mg, range 20–240 mg), but no difference between the treatment groups was noted in stratum B (265 ± 523 mg, range 0–1857 mg, versus 223 ± 316 mg, range 0–1070 mg). However, in stratum A more positive 7-day fluid balances were observed in the AP301 group including three patients with severe burn injury than in the placebo group. The number of patients receiving corticosteroids was 14 in each treatment group (three per group in stratum A and 11 per group in stratum B). Patients in the AP301 group received higher noradrenalin doses at screening in both strata (Table 2).

**Table 7:** Adverse events (*n* > 2)

Adverse event	AP301 (*n* = 20)	Placebo (*n* = 20)	Total (*n* = 40)
Tracheostomy	5 (25)	8 (40)	13 (32.5)
Anemia	3 (15)	6 (30)	9 (22.5)
Worsening of existing anemia	5 (25)	2 (10)	7 (17.5)
Cardiac arrest	5 (25)	1 (5)	6 (15)
Fever	1 (5)	4 (20)	5 (12.5)
Thrombopenia	3 (15)	1 (5)	4 (10)
Leucocytosis	3 (15)	1 (5)	4 (10)
Atrial flutter/fibrillation	1 (5)	3 (15)	4 (10)
Pleural effusion	0 (0)	3 (15)	3 (7.5)

Data are presented as *n* (%)

## Discussion

This randomized placebo-controlled phase IIa trial is the first clinical trial reporting the use of inhaled AP301 in mechanically ventilated patients with ARDS. The total number of adverse events was no different between the treatment groups. In addition, all adverse events could be explained by the course of the underlying diseases and the severity of the critical illness.

The comparison of primary and secondary endpoints between the treatment groups did not differ in this small phase IIa study. An exploratory post-hoc subgroup analysis indicated reduced EVLWI in patients with SOFA scores ≥11 receiving treatment with inhaled AP301.

We stratified patients prior to randomization by SOFA score to ensure equal distribution of patients according to the severity of the underlying disease to the both treatment arms. With this approach we aimed to avoid an uneven distribution of severity of extrapulmonary organ failure between the treatment groups due to variances in severity of illness. A cut-off value of SOFA score ≥11 was chosen to reflect the increased in-hospital mortality at SOFA scores ≥11 [33, 34].

To better understand if treatment response differed in patients with distinct severity of illness, we performed a post-hoc exploratory subgroup analysis of the SOFA score strata. We observed a reduction in EVLWI and ventilation pressures over 7 days together with a trend of more ventilator-free days in patients with initial SOFA scores ≥11 who received AP301. No treatment effect of AP301 was observed in patients with SOFA scores ≤10 at screening. These differences between the patients stratified according to severity of illness (SOFA score strata) may have several explanations. Owing to a small sample size, less severely ill patients (stratum A) may have had a profile that precluded a benefit from AP301. By chance, all three patients with burn injuries in the study were randomized to AP301 treatment in stratum A. Two of these patients did not exhibit a reduction in EVLWI over 7 days of treatment. Another explanation for the absence of a treatment effect in stratum A may be that patients with less severe critical illness might present with transient reductions in arterial oxygenation that are easily reversed and that are not due to pulmonary inflammation, resulting in AP301 ineffectiveness.

Due to the small sample size, uneven distribution of severity of disease could result in different response to treatment. For instance, at screening, more severely ill patients (stratum B, SOFA scores ≥11) had more ECMO therapy, prone positioning, and higher EVLWI values.

Nonhydrostatic pulmonary edema is an important characteristic of ARDS that originates from the increased permeability of the alveolar-capillary barrier in conjunction with impaired AFC [6, 9, 35–37]. The peptide AP301 activates ENaCs by increasing their open state probability and expression, even in the presence of bacterial toxins [12, 14, 15, 38]. The former occurs through strengthening of the complex formation between ENaC and its chaperone protein MARCKS, the latter occurs by means of blunting ubiquitination and degradation of the ENaC subunits [23]. Of note, in contrast to drugs such as β_2_ adrenergic agonists, which activate ENaCs in a cAMP-dependent manner, AP301 directly activates ENaCs upon binding to their α subunit [15]. Apart from activating AFC, AP301 also strengthens capillary barrier function in human lung MVEC as well as in rodent models of pneumonia [20]. In a variety of animal models of ARDS, pulmonary administration of AP301 substantially alleviated pulmonary edema [16–22, 39]. The present study demonstrated for the first time that AP301 inhalation may reduce EVLWI in severely ill patients with ARDS (SOFA scores ≥11), while the causality of this finding needs further investigation. We hypothesize that AP301 might provide more benefit in those patients that display both impaired AFC and increased capillary leak, which would particularly be the case for patients with direct lung injury, i.e., with severe pneumonia. We have actually detected that the dose of AP301 necessary to resolve pulmonary edema decreases in models combining both capillary leak and impaired AFC [20, 23, 40], as compared to models of hydrostatic edema in which only AFC dysfunction occurs [16].

Although fluid management was left to the discretion of the treating physicians and we did not use a standardized treatment protocol, the observed effects on EVLWI could not be explained by differences in fluid balance.

Our study has several limitations. The treatment intervention was added to standard of care for patients with ARDS at the Vienna General Hospital. Protocols for ventilation and weaning or handling of diuretic therapy were not strictly defined in the study protocol, and the treatment depended on the decisions of treating physicians and staff who were by design not involved in conduct of the study. Another major limitation is that this small study included a heterogeneous population of patients with ARDS caused by various clinical conditions, and it was not adequately powered to identify differences in important outcome parameters such as length of ICU stay or survival.

Besides one AE (episode of decreased tidal volume during mechanical ventilation) after AP301 inhalation, no safety concerns attributable to the study drug were observed. Most AEs represent common complications in critically ill patients with ARDS, or they were caused by underlying diseases or preexisting comorbidities, precluding a relationship to the study therapy. Mortality did not differ between the treatment groups, peaking at 30% in the AP301 group, which is the observed mortality rate of ARDS according to the literature [1, 2]. Of note, two patients with 60% burn injury, which is associated with an even higher mortality rate [41, 42], were included in the AP301 group. Considering all details of the patients’ case histories, the five fatal cardiac arrests in the AP301 group were not related to the study therapy. Three of these patients died following the decision to limit intensive care support due to futility. The causes of cardiac arrest in the other two patients were myocardial infarction and multiorgan failure in a patient with 60% burn injury, respectively. Only one cardiac arrest occurred within the active treatment period, but study treatment was stopped 2 days before the event owing to the patient’s unstable condition.

Of note, in a large clinical trial of the ARDS network, 79% of patients who died had a “do not resuscitate” order [43]. In the present study, 50% of patients who died within 28 days had an order to “allow natural death,” including four patients in the AP301 group and one patient in the placebo group.

## Conclusion

In conclusion, treatment with inhaled AP301 did not reduce EVLWI and appeared to be safe in patients with mild to severe ARDS. An exploratory post-hoc subgroup analysis of SOFA strata indicated reduced EVLWI and ventilator pressures in patients with SOFA scores ≥11. These results need further clarification in upcoming multicenter clinical trials.

## Additional file

Additional file 1: The study protocol as approved by the Ethics Committee. (PDF 924 kb)

## Abbreviations

AE: Adverse event
AFC: Alveolar fluid clearance
ARDS: Acute respiratory distress syndrome
CPR: Cardiopulmonary resuscitation
ECMO: Extracorporeal membrane oxygenation
ENaC: Epithelial sodium channel
EVLWI: Extravascular lung water index
GOCA: Gas exchange, organ failure, cause, and associated conditions
ICU: Intensive care unit
IQR: Interquartile range
LIS: Lung injury score
MVEC: Microvascular endothelial cell
PBW: Predicted body weight
PEEP: Positive end-expiratory pressure
PiCCO: Pulse contour analysis and continuous cardiac output
SAE: Serious adverse event
SD: Standard deviation
SOFA: Sequential organ failure assessment
TNF: Tumor necrosis factor
