# Supplementary material for: Inhaled AP301 for treatment of pulmonary edema in mechanically ventilated patients with acute respiratory distress syndrome: a phase IIa randomized placebo-controlled trial
Source: Crit Care. 2017 Jul 27;21:194. doi: 10.1186/s13054-017-1795-x (PMC5531100; doi:10.1186/s13054-017-1795-x)
Supplement: Additional file 1: — The study protocol as approved by the Ethics Committee. (PDF 924 kb) [file 13054_2017_1795_MOESM1_ESM.pdf]

CLINICAL STUDY PROTOCOL

Proof of concept study in male and female intensive care patients to investigate the clinical effect of repetitive orally inhaled doses of AP301 on alveolar liquid clearance in acute lung injury

AP301-II-001

Version 1.2 / 18.06.2012

Confidentiality Statement
The information contained in this document, especially unpublished data, is the property of the sponsor of this study. It is therefore provided to you in confidence as an investigator, potential investigator, or consultant, for review by you, your staff, and an Independent Ethics Committee or Institutional Review Board. It is understood that this information will not be disclosed to others without written authorization from the sponsor of the study except to the extent necessary to obtain informed consent from those persons to whom the study drug may be administered.

**Test drug (IMP) and**	AP301 25mg powder for reconstitution for solution for inhalation
**Pharmaceutical Company**	APEPTICO Forschung und Entwicklung GmbH<br>c/o mingo bueros<br>Mariahilfer Straße 136, Top 1.15<br>1150 Vienna, Austria
**Protocol authors**	Univ.-Doz. Dr. Bernhard Fischer (APEPTICO Forschung und Entwicklung GmbH, Mariahilfer Straße 136, Top 1.15, 1150 Vienna, Austria);<br><br>Dr. Helmut Pietschmann (APEPTICO Forschung und Entwicklung GmbH, Mariahilfer Straße 136, Top 1.15, 1150 Vienna, Austria);<br><br>Priv.-Doz. Johannes Pleiner (Koordinierungszentrum für klinische Studien, Borschkegasse 8b/E6, 1090 Vienna, Austria);<br><br>Dr. Günther Nirnberger (Bioconsult GmbH, Fritz Konir-Gasse 19, 2384 Breitenfurt, Austria);<br><br>Dr. Robert Hermann (Clinical research appliance Rossittenstrasse 15, 78315 Radolfzell, Germany)
**Investigator**	Prof. Dr. Roman Ullrich
**Document type**	Clinical study protocol
**Study phase**	Phase IIa
**Document status**	Protocol Version 1.2
**Date**	18.06.2012
**Number of pages**	56

1. SPONSOR, ADVISOR, INVESTIGATOR, MONITOR, STATISTICIAN, DATA MANAGEMENT AND SIGNATURES

Sponsor (AMG §§ 2a, 31,32)
APEPTICO Forschung und Entwicklung GmbH
Mariahilfer Straße 136, Top 1.15
1150 Vienna, Austria

Representatives: Univ.-Doz. Dr. Bernhard Fischer (CEO)

__________________ ____________

Signature Date

Dr. med. Helmut Pietschmann

__________________ ____________

Signature Date

Investigator (AMG §§ 2a, 35,36)

Ao. Univ.-Prof. Dr. Roman Ullrich
Department of Anesthesia, General Intensive Care and Pain Control
Medical University of Vienna
Währinger Gürtel 18-20
1090 Vienna, Austria

__________________ ____________

Signature Date

Monitor / CRO (AMG §§ 2a, 33,34)

Koordinierungszentrum für klinische Studien (KKS)
Borschkegasse 8b/E6
1090 Vienna, Austria

Representative: PD Dr. Johannes Pleiner

___________________ ____________
Signature Date

Statistician

Bioconsult GmbH
Fritz Konir-Gasse 19
2384 Breitenfurt, Austria

Representative: Dr. Günther Nirnberger

___________________ ____________
Signature Date

Clinical Trials Centers:

Medical University of Vienna
Department of Anesthesia, General Intensive Care and Pain Control
Währinger Gürtel 18-20
1090 Vienna, Austria

2. STATEMENT OF PRINCIPAL INVESTIGATOR

I have read the preceding protocol entitled "*Proof of concept study in male and female intensive care patients to investigate the clinical effect of repetitive orally inhaled doses of AP301 on alveolar liquid clearance in acute lung injury*" and agree to follow the instructions laid down in this protocol and to conduct this study in accordance with procedures intended to ensure adherence to the requirements of current Good Clinical Practice (GCP), with the Declaration of Helsinki and with the laws and regulations of the country in which the study takes place. Moreover the clinical study site will keep all information obtained from the participation in this study confidential unless otherwise agreed in writing.

AO. Univ.-Prof. Dr. Roman Ullrich (Principal Investigator)

____________________ _____________________________

Date Signature

3. PROTOCOL SYNOPSIS

TITLE	*Proof of concept study in male and female intensive care patients to investigate the clinical effect of repetitive orally inhaled doses of AP301 on alveolar liquid clearance in acute lung injury.*					
OBJECTIVES	**Primary Objective**<br>• *To assess the effect of orally inhaled AP301 on alveolar liquid clearance in ALI patients with the purpose to assess the treatment associated changes of extravascular lung water (EVLW) within 7 days of treatment.*<br>**Secondary Objectives**<br>*To assess:*<br>• *the treatment associated changes of oxygenation as measured by the PaO_2 / FiO_2 ratio until day 7 of therapy,*<br>• *the ventilatory plateau pressure and other ventilation parameters (Tidal volume, positive endexpiratory pressure, FiO2, respiratory rate, PIP, mean airway pressure and peak airway pressure) until day 7 of therapy,*<br>• *the Murray lung injury score (a composite variable that includes components of oxygenation, compliance, positive end-expiratory pressure, and the appearance of the chest radiograph),*<br>• *the duration of stay at ICU in the first 28 days,*<br>• *the duration of controlled ventilation period until extubation,*<br>• *the number of days free from ventilatory support [for > 48 h] in the first 28 days*<br>• *the survival status at day 28,*<br>• *the local and systemic safety and tolerability and to identify possible dose related adverse events.*					
DESIGN / PHASE	*Interventional, randomized, double-blind, placebo-controlled, parallel-group, monocentric phase II study*					
STUDY PLANNED DURATION	**First patient First visit**	*3Q 2012*	**Last patient First visit**	*3Q 2013*	**Last patient Last visit**	*3Q 2013*
CENTER(S) / COUNTRY(IES)	*1 centre in Austria*					
PATIENTS / GROUPS	*40 patients in 2 groups*<br>*20 patients per group*<br>*Randomization ratio 1:1, stratification(SOFA score ≤ 10, SOFA score ≥ 11)*					
INCLUSION CRITERIA	- *age ≥ 18*<br>- *intubated and mechanically ventilated male and female patients of the Intensive Care Units of the Department of Anesthesia, General Intensive Care and Pain Control*<br>- *meets the criteria of ALI (as defined by the American-European Consensus Conference on ALI/ ARDS):*<br>- *Onset of ALI within 48 hours*					

	- *Bilateral infiltrates seen on frontal chest radiograph*<br>- *PCWP ≤ 18 mm Hg or no clinical evidence of left atrial hypertension*<br>- *paO_2/ FiO_2 ratio ≤ 300 mm Hg*<br>- *EVLW in PiCCO® at screening ≥ 8 ml/PBW*<br>- *Meeting criteria for extensive hemodynamic monitoring according to investigators discretion*<br>- *ICU Patients being mechanically ventilated and are stable in this condition for at least 8 hours*<br>- *Negative pregnancy test and adequate contraception in female patients of childbearing potential*<br>- *Informed consent:*<br>- *For patients that are temporarily unable to consent (e.g. comatose patients) a subsequent informed consent has to be provided.*
EXCLUSION CRITERIA	*Medical Issues*<br>• *History of clinically relevant allergies or idiosyncrasies to AP301 or any other inactive ingredient(s) of the investigational product*<br>• *Brainstem death at screening*<br>• *Current evidence of septic shock as defined by the Surviving Sepsis Campaign (Presence of acute organ dysfunction secondary to infection plus hypotension with systolic blood pressure < 90 mm Hg or mean arterial pressure (MAP) < 70 mm Hg for not less than one hour which cannot be reversed with fluid resuscitation)*<br>• *Neutrophil count <0.3 x 10^9 L*<br>• *Patients under immunosuppression: high dose steroids (> 80 mg Prednisolone /d; > 300 mg hydrocortisone / d), cancer treatment (chemotherapy or biological) or therapy with other immunosuppressive agents for organ transplantation within 2 weeks*<br>• *BMI < 18.5 or > 35*<br>• *Cardiogenic pulmonary edema diagnosed by echocardiography or pulmonary artery catheter*<br>• *Pregnancy / lactation or intention to fall pregnant during the time course of the study*<br>• *Women of childbearing potential as well as men of procreative capacity who are not using adequate contraception*<br><br>*General Issues*<br>• *Participation in other interventional drug trials*
STUDY PERIODS	• *1 year*
INVESTIGATIONAL DRUG	***AP301 25 mg powder for reconstitution for solution for inhalation:***<br>*dose:*<br>*orally delivered doses of 87.6 mg AP301 (dose per subject, 5 ml nebulizer filling dose) are inhaled every 12 hours for a total of 7 days*<br><br>*administration:*

	oral inhalation via nebulizing device (Aeroneb® solo nebulizing system)<br><br>*manufacturer:* Aptuit Glasgow, UK
COMPARATIVE DRUG /CONTROL CONDITION	***Placebo solution:***<br>*dose:*<br>*placebo solution (0.9 % physiologic NaCl, 5 ml nebulizer filling dose) is inhaled every 12 hours for a total of 7 days*<br><br>*administration:*<br>*oral inhalation via nebulizing device (Aeroneb® solo nebulizing system)*<br><br>*manufacturer:* B. Braun Melsung AG, Germany (commercial product)
CONCOMITANT MEDICATION	**The following concomitant medications are not allowed:**<br><br>*Immunosuppressives: high dose steroids (> 80 mg Prednisolone /d; > 300 mg hydrocortisone / d), cancer treatment (chemotherapy or biological) or therapy with other immunosuppressive agents for organ transplantation*
EFFICACY ENDPOINTS	***Primary:***<br>• *EVWL (measured with PiCCO® technique) until day 7 of treatment*<br>***Secondary:***<br>• *Oxygenation index (PaO_2 / FiO_2 ratio) until day 7 of treatment*<br>• *Ventilator plateau pressure until day 7 of treatment*<br>• *Murray Lung Injury Score until day 7 of treatment*<br>• *Ventilation parameters / lung function: tidal volume (V_t), positive endexpiratory pressure (PEEP), respiratory rate, FiO_2, PIP, mean airway pressure, peak airway pressure until day 7 of treatment*<br>• *Duration of stay at ICU in the first 28 days*<br>• *Duration of controlled ventilation period until extubation*<br>• *Number of days free from ventilation support (for > 48 h) in the first 28 days*<br>• *Survival status at day 28*
TOLERABILITY / SAFETY ENDPOINTS	*The local and systemic safety and tolerability and to identify possible dose related adverse events:*<br>• *Hemodynamic monitoring: heart rate (HR), systolic BP, mean arterial pressure (MAP), PBV, 24 hours fluid balance, CI and cardiac output (measured by PiCCO®) until day 7 of treatment*<br>• *Vital signs: pulse rate, and respiratory rate until day 7 of treatment*<br><br>*(Presence/absence/severity and duration of adverse effects – number and severity of adverse events (AEs), serious adverse events (SAEs) and suspected unexpected serious adverse events (SUSARs))*
STATISTICAL METHODOLOGY	**Primary Endpoint**<br>*Primary Endpoint is EVWL (measured with PiCCO® technique) between day 0 and day 7 of treatment.*<br>*Statistical tests will be two-tailed at the 5% significance level (Power*

	80%). The primary efficacy variable (EVWL) will be analyzed confirmatory between the two conditions (AP301 vs. Placebo) using an ANCOVA (analysis of covariance) model or the non parametric Mann-Whitney U-test, dependant on the distribution of data.<br><br>**Null and alternative hypotheses:**<br>*H_0: Mean EVWL (AUC between Day 0 and Day 7) in the AP301 group = Mean EVWL (AUC between Day 0 and Day 7) in the Placebo group*<br><br>*H_1: Mean EVWL (AUC between Day 0 and Day 7) in the AP301 group < Mean EVWL (AUC between Day 0 and Day 7) in the Placebo group*<br><br>**Secondary Endpoints**<br>*Secondary efficacy variables and exploratory variables will be presented using appropriate descriptive methods, and will be analysed in an explorative sense. Statistical tests and the p-values attached to them will be regarded as descriptive and not as tests of hypotheses.*<br><br>**Sample size calculation**<br>*This study is intended to show the superiority of AP301 vs. Placebo, using "EVLW" (AUC between Day 0 and day 7) as Primary Efficacy Variable.*<br>*A mean difference of at least 40% between the two treatment conditions (AP301 vs. Placebo) will be supposed for EVLW (baseline SD about 40 %), indicating statistically significant reduction of EVLW by use of AP301 compared to Placebo.*<br>*A sample size of 40 (20 / group) will be sufficient to show a statistically significant difference between Verum and Placebo with $p< 0.05$ (two sided) and a power of 80%.*

4. LIST OF ABBREVATIONS

ICH	International Conference on Harmonization
GCP	Good Clinical Practice
DOH	Declaration of Helsinki
EVLW	extravascular lung water
ICU	Intensive Care Unit
ALI	Acute lung injury
PaO_2	arterial oxygen tension
FiO_2	fraction of inspired oxygen
VALI	ventilator associated lung injury
ARDS	acute respiratory distress syndrome
LIRI	lung ischemia-reperfusion injury
SOFA	Sepsis-related Organ Failure Assessment
GOCA	Gas exchange, organ failure, cause, and associated conditions
PCWP	pulmonary capillary wedge pressure
MAP	mean arterial pressure
BMI	body mass index
V_t	tidal volume
PEEP	positive endexpiratory pressure
ENaC	amiloride-sensitive epithelial sodium ion channel
TNF	tumour necrosis factor
BP	blood pressure
sBP	systolic blood pressure
CI	cardiac index
PBV	pulmonary blood volume
WFI	water for injection
PIP	peak inspiratory pressure
PBW	predicted body weight

5. TABLE OF CONTENTS

CLINICAL STUDY PROTOCOL 1

1. SPONSOR, ADVISOR, INVESTIGATOR, MONITOR, STATISTICIAN, DATA MANAGEMENT AND SIGNATURES 3

2. STATEMENT OF PRINCIPAL INVESTIGATOR 5

3. PROTOCOL SYNOPSIS 6

4. LIST OF ABBREVATIONS 10

5. TABLE OF CONTENTS 11

6. BACKGROUND INFORMATION 16

6.1 BACKGROUND 16
6.2 NON-CLINICAL STUDIES 17
6.3 CLINICAL STUDIES 18
6.4 STUDY RATIONALE 19

7. STUDY OBJECTIVES 20

7.1 PRIMARY OBJECTIVE 20
7.2 SECONDARY OBJECTIVES 20

8. STUDY DESIGN 20

8.1 STUDY POPULATION 22
8.1.1 SUBJECT POPULATION 22
8.1.2 INCLUSION CRITERIA 22
8.1.3 EXCLUSION CRITERIA 23
8.1.4 FEMALES OF CHILDBEARING AGE 23
8.1.5 STUDY DURATION 24
8.1.6 WITHDRAWAL AND REPLACEMENT OF SUBJECTS 24
8.1.7 PREMATURE TERMINATION OF THE STUDY 25

9. METHODOLOGY 25

9.1 STUDY MEDICATION 25
9.1.1 DOSAGE AND ADMINISTRATION 26
9.1.2 STUDY DRUG INTERRUPTION OR DISCONTINUATION 27
9.1.3 STUDY DRUG PREMATURE PERMANENT DISCONTINUATION 27
9.1.4 STUDY DRUG PACKAGING, LABELING AND DELIVERY 27
9.1.5 IMP ADMINISTRATION & HANDLING 28
9.1.6 DRUG ACCOUNTABILITY 29
9.1.7 PROCEDURES TO ASSESS SUBJECTS COMPLIANCE 30
9.1.8 CONCOMITANT MEDICATION 30
9.2 RANDOMIZATION AND STRATIFICATION 30
9.3 BLINDING 31
9.3.1 EMERGENCY PROCEDURE FOR UNBLINDING 31
9.3.2 UNBLINDING AT THE END OF THE STUDY 31
9.4 BENEFIT AND RISK ASSESSMENT 31
9.5 STUDY PROCEDURES 32
9.5.1 GENERAL RULES FOR TRIAL PROCEDURES 32
9.5.2 SCREENING INVESTIGATION (DAY 0) 33
9.5.3 TREATMENT PHASE (DAY 1 TO 7) 33
9.5.4 END-OF-STUDY (EOS) INTERVIEW (DAY 28 + 7 DAYS) 34
9.5.5 EARLY TERMINATION (ET) 34
9.5.6 METHODS OF EVALUATION 34

10. SAFETY DEFINITIONS AND REPORTING REQUIREMENTS 37

10.1 AVERSE EVENTS (AES) 37
10.1.1 SUMMARY OF KNOWN AND POTENTIAL RISKS OF THE STUDY DRUG 37
10.1.2 DEFINITION OF ADVERSE EVENTS 38
10.2 SERIOUS ADVERSE EVENTS (SAES) 39
10.2.1 HOSPITALIZATION – PROLONGATION OF EXISTING HOSPITALIZATION 39
10.2.2 SAES RELATED TO STUDY-MANDATED PROCEDURES 40
10.2.3 SUSPECTED UNEXPECTED SERIOUS ADVERSE REACTIONS (SUSARS) 40
10.2.4 PREGNANCY 40
10.3 SEVERITY OF ADVERSE EVENTS 41
10.4 RELATIONSHIP TO STUDY DRUG 42
10.5 REPORTING PROCEDURES 42
10.5.1 REPORTING PROCEDURES FOR SAES 43
10.5.2 REPORTING PROCEDURES FOR SUSARS 44
10.5.3 ANNUAL SAFETY REPORT 44
10.5.4 SAFETY MONITORING BOARD 45

11. FOLLOW-UP 45

11.1 FOLLOW-UP OF STUDY PARTICIPANTS INCLUDING FOLLOW-UP OF ADVERSE EVENTS 45
11.2 TREATMENT AFTER END OF STUDY 45

12. STATISTICAL METHODOLOGY AND ANALYSIS 45

12.1 ANALYSIS SETS 46
12.2 SAMPLE SIZE CONSIDERATIONS 46
12.3 RELEVANT PROTOCOL DEVIATIONS 46
12.4 STATISTICAL ANALYSIS PLAN 47
12.5 MISSING, UNUSED AND SPURIOUS DATA 47
12.6 ENDPOINTS ANALYSIS 47
12.6.1 PRIMARY ENDPOINT ANALYSIS 47
12.6.2 SECONDARY ENDPOINT ANALYSIS 48
12.6.3 SAFETY AND TOLERABILITY ENDPOINTS 48
12.6.4 BASELINE PARAMETERS AND CONCOMITANT MEDICATIONS 49
12.7 INTERIM ANALYSIS 49
12.8 SOFTWARE PROGRAM(S) 49

13. DOCUMENTATION AND DATA MANAGEMENT 49

13.1 DOCUMENTATION OF STUDY RESULTS 49
13.1.1 CASE REPORT FORM (CRF) 50
13.1.2 DATA COLLECTION 50
13.1.3 IDENTIFICATION DATA TO BE CONSIDERED AS SOURCE DATA 50
13.2 SAFEKEEPING 50
13.3 QUALITY CONTROL AND QUALITY ASSURANCE 51
13.3.1 PERIODIC MONITORING 51
13.3.2 AUDIT AND INSPECTIONS 52
13.4 REPORTING AND PUBLICATION 52
13.4.1 PUBLICATION OF STUDY RESULTS 52

14. ETHICAL AND LEGAL ASPECTS 52

14.1 INFORMED CONSENT OF SUBJECTS 52
14.2 ACKNOWLEDGEMENT / APPROVAL OF THE STUDY 53
14.2.1 CHANGES IN THE CONDUCT OF THE STUDY 53
14.3 INSURANCE 54
14.4 CONFIDENTIALITY 54
14.5 ETHICS AND GOOD CLINICAL PRACTICE (GCP) 54

15. REFERENCES 56

16. TABLES 56

Table 1: VISIT AND ASSESSMENT SCHEDULE

Assessment	SCREENING	TREATMENT *7 days*							FOLLOW-UP[12)] *28th day/ +7 days*
	Screening Day 0	*Treatment Day 1*	*Treatment Day 2*	*Treatment Day 3*	*Treatment Day 4*	*Treatment Day 5*	*Treatment Day 6*	*Treatment Day 7*	*End of Study Day 28*
**Informed Consent[1)]**	X								
**Inclusion / Exclusion Criteria**	X								
**Randomization**		X							
**Medical History[2)]**	X								
**Concomitant/change in medication**	X	X	X	X	X	X	X	X	X
**Demography[3)]**	X								
**Vital Signs[4)]**	X	X	X	X	X	X	X	X	
**Clinical Laboratory[5)]**	X	X	X	X	X	X	X	X	
**PiCCO®: EVLW[6)]**	X	X	X	X	X	X	X	X	
**GOCA**	X								
**Murray Lung injury score**	X	X	X	X	X	X	X	X	
**SOFA**	X								
**Hemodynamic parameters[7)]**	X	X	X	X	X	X	X	X	
**Pregnancy Test**	X								
**Study Treatment[8)]**		X	X	X	X	X	X	X	
**Ventilation parameters[9)]**	X	X	X	X	X	X	X	X	
**Days until extubation[10)]**									X
**Survival**									X
**Adverse Events[11)]**		X	X	X	X	X	X	X	X

1) For patients who are temporarily unable to consent, a subsequent informed consent has to be provided

2) The value of the Glasgow Coma Scale will be assessed retrospectively

3) Age, ethnicity, height, weight, gender

4) Pulse rate, respiratory rate as well as temperature (assessed only at screening)

5) Haematology & Chemistry, including arterial blood gases

[6)] EVLW values will be assessed every 12 hours until day 7 (+/- 30 minutes). EVLW must be measured within 60 to 120 minutes after inhalation of IMP.

[7)] Haemodynamic parameters: HR, MAP, PBV, systolic BP, 24 hours fluid balance, cardiac output and cardiac index (CI) as measured by PiCCO®

[8)] Study drug will be administered every 12 hours (+/- 30 minutes)

[9)] Ventilation parameters: plateau pressure, tidal volume, endexpiratory pressure (PEEP), respiratory rate, FiO_2, mean airway pressure, peak airway pressure, PIP

Ventilation settings are only assessable until extubation

[10)] Furthermore, the duration of stay at ICU and number of days free from ventilator support (for > 48h) will be documented

[11)] AEs prior administration of IMP will be documented on the Medical History page of the eCRF.

[12)] The follow-up information will be assessed via telephone

6. BACKGROUND INFORMATION

6.1 Background

The pulmonary permeability oedema is characterized by a reduced alveolar liquid clearance capacity, combined with an endothelial/epithelial hyper-permeability. Two separate barriers form the alveolar–capillary barrier: (a) the microvascular endothelium and (b) the alveolar epithelium. Both endothelial and epithelial injury and increased vascular permeability lead to the formation of the pulmonary permeability oedema in the life-threatening condition Acute Lung Injury. There are many possible causes of ALI, such as inhaling high concentrations of smoke, toxins, or oxygen; severe burns; blood infections; lung infections; or trauma to other parts of the body.

Acute Lung Injury is a condition in which the capacity of the lungs to oxygenate the blood is greatly reduced, even if oxygen is administered in high concentrations. The lungs are composed of tiny structures called alveoli. It is in the alveoli where oxygen and carbon dioxide are exchanged between the air that is inhaled and the blood that perfuses the lungs in tiny vessels called capillaries. When the lungs are injured and / or the surrounding capillaries are injured, blood and fluids begin to leak from the capillaries, into the alveoli. Subsequently, air cannot enter the alveoli and pass into the blood, which means that the normal functions of the lung tissue are impaired. This will lead to inflammation (a response to the injury caused to the tissue that includes swelling) and progressive formation of scar tissue in the walls of the alveoli. Without treatment, the gas exchange derangement and abnormal lung mechanics can result in progressive hypoxemia, respiratory muscle fatigue, and eventual respiratory arrest and death.

For Acute Lung Injury and the Acute Respiratory Distress Syndrome mortality rates of 30% to 60% have been reported (Review in: (Ware & Matthay, 2000)).

In patients with ALI there exists a close correlation between mortality and the capacity of the patients to resolve the pulmonary oedema. Maximal alveolar fluid clearance is associated with better clinical outcomes (Ware & Matthay, 2001).

Alveolar liquid clearance from the alveolus into the interstitium is mainly based on an active vectorial sodium transport, largely through the highly regulated apical, amiloride-sensitive epithelial sodium channel complex (ENaC), with concomitant passive water transport. The amiloride-sensitive epithelial sodium channel complex (ENaC) is expressed in cuboidal type II alveolar epithelial cells which make approx. 10% of the alveolar surface area. The basolaterally expressed, ouabain-inhibitable Na^+,K^+-ATPase then further drives the vectorial transport into the interstitium and,

ultimately, into the lymphatic and blood vessels. These transport processes are impaired by the pulmonary permeability oedema as present in ALI.

Accordingly, a high expression rate of functionally active amiloride-sensitive epithelial sodium channel complex (ENaC) in type II alveolar cells is essential for liquid clearance from alveoli to restore normal gas exchange.

Today, Acute Lung Injury has no specific treatment. Mechanical ventilation aims to correct systemic oxygen level, but may induce so-called “Ventilation Induced Lung Injury”. Mechanical ventilation does not resolve the pulmonary oedema and does not support cell and tissue repair to minimise fluid leakage from injured blood capillaries into the alveoli. Fluid and haemodynamic management supports an acute venodilatory response with a reduction in preload that may help to relieve pulmonary congestion in addition to the more delayed diuretic response, but it fails to prevent oedema formation / oedema resolution and it does not interfere with tissue damage. Diuretics are known to cause several side-effects, such as hypotension, diminished renal perfusion, renal failure, hypokalemia and hypomagnesemia, metabolic alkalosis, carbohydrate intolerance, hyperuricemia and hypersensitivity. Vasodilators may lead to relaxation of the muscular wall of the vessels and as such affect the relationship between mean arterial pressure, cardiac output and total peripheral resistance. However, all these functions are only of symptomatic nature and do not “treat” the lung injury. In addition, venous vasodilators given to stabilise cardiac function are associated with the risk of precipitating ventricular arrhythmias. Steroid hormones, such as glucocorticoids, if administred systemically may reduce inflammation reaction in the lung tissue, but on the other hand increase the incidence of infection. Repeated application of glucocorticoids may result in resistance and serious side-effects, such as immunosuppression, hyperglycemia, reduced bone density, adrenal insufficiency, increased urea formation and other. Although effective in infants with the neonatal respiratory distress syndrome, surfactant replacement therapy is of less value in Acute Lung Injury. Both the application of the surfactant by instillation and the delivery to the distal air spaces are problematic in adult patients with Acute Lung Injury.

6.2 Non-clinical Studies

Non-clinical studies in cell-based and animal models revealed following essential pharmacological properties of the AP301 peptide:

- AP301 activates Alveolar Fluid Clearance (ALC).

- AP301 restores pulmonary gas exchange.

- AP301 produces a progressive recovery of dynamic lung compliance and airway resistance.

- AP301 prevents and restores hyper-permeability of connecting cells and tissues in the lung.

- AP301 prevents Ischemia Reperfusion Injury in transplanted lungs.

The bio-activity of the AP301 peptide results from following molecular reactions:
- AP301 activates the amiloride-sensitive epithelial sodium channel complex (ENaC) in human epithelial and endothelial lung cells.

- AP301 protects the endothelial / epithelial barrier function from virulence factors of common lung pathogenic bacteria by decreasing intracellular levels of Reactive Oxygen Species (ROS), by regulating Protein Kinase C (PKC) activation and Myosin Light Chain (MLC) phosphorylation and by restoring the RhoA/Rac ratio.

The standard battery of safety pharmacology studies did not reveal drug-related adverse effects in any of the animal models.From animal studies in various species (rodent and non-rodent) it has become evident that peptide clearance from blood is quite fast (decrease by 3 orders of magnitude within 2 hours) after bolus application of 25 mg AP301/kg BW via intravenous injection. In the 14 day toxicity study in the rat, lung tissue samples were taken after the last treatment on day 14 and analysed for presence of AP301. As a result, no test item was detected in lung tissue approximately 30, 60 and 90 minutes after the end of treatment in the low, mid and high dose group respectively.

For further information regarding non-clinical studies (efficacy/safey/toxicology) please refer to the Investigators Broschure).

6.3 Clinical Studies

So far AP301 has been investigated in phase I in healthy volunteers. To investigate the safety and tolerability of orally inhaled AP301 a randomised, double-blind, placebo-controlled, sequential single-dose-escalation first-in-man study has been performed at the Vienna General Hospital between May 2012 and October 2012. Forty-eight healthy male subjects were exposed to single doses of either a nebulised liquid formulation of AP301 for oral inhalation or a nebulised liquid formulation of saline as control. AP301 was administered in six ascending orally delivered doses of 4.32, 12, 30, 60, 90 and 120 mg per subject.
The safety and tolerability profile of the compound was excellent. AP301 was generally well tolerated. No Serious Adverse Events (SAE) or unexpected AEs (SUSARs) occurred throughout the

study. In total 15 mild adverse events (AEs) were reported by 11 out of 48 subjects, of which 6 AEs were judged to be possibly related to single dose of AP301 or matching placebo (see Table 2.)
The safety and tolerability profile of the compound was excellent. For the proposed phase IIa clinical study one dose level of 88 mg of orally delivered AP301 per subject will be used (based on 125 mg nebuliser filling dose). This dose level is well below the highest dose level used in the phase I study. For comparison a control group with placebo treatment (saline solution) is planned. Based on results of pre-clinical studies as well as the phase I clinical study no systemic exposure of AP301 is expected during the planned phase IIa clinical study. AP301 has been designed to activate an ion-channel, located at the luminal side of the alveoli. For this reason an aqueous formulation of AP301 is nebulised and inhaled in order to reach its target. AP301 has not been designed to pass epithelial and endothelial barriers.

6.4 Study rationale

Today, Acute Lung Injury has no specific treatment, the condition is rare and considered as Orphan Condition in the European Community.

The mainstay of treatment is supportive care, mainly to avoid iatrogenic complications and treat the underlying cause, while maintaining adequate oxygenation. It appears that there does not exist a specific single treatment approach to this condition. Apparently, a commonly accepted method or consensus among clinicians has not been established.

The therapeutic potential of AP301 to resolve the pulmonary permeability oedema has been high-lighted in Editorials in Critical Care Medicine (Matthay, 2008) and Vascular Pharmacology (Black, 2010). If compared to other investigational medicinal products, AP301 represents a new molecular type and new concept for prevention and treatment of hyper-permeability, treatment of pulmonary permeability oedema and prevention of ischemia reperfusion injury. The test compound - for the first time - represents a molecule that directly assists in alveolar liquid clearance and that counter-acts Reactive Oxygen Species as well as bacterial and viral virulence factors in the lung tissue. AP301 peptide is expected to reduce the leakage of blood and fluids from the capillaries in the lungs (hyper-permeability) and to activate alveolar liquid clearance in patients suffering from Respiratory Failure and pulmonary permeability oedema. It is expected to improve oxygenation of patients. Both, alveolar fluid clearance and restoration of endothelial-epithelial barrier function are associated with better clinical outcomes of the life-threatening condition “Acute Lung Injury”.

This interventional, randomized, double-blind, placebo-controlled, parallel-group, monocentric study aims to investigate the clinical effect and the safety of orally inhaled AP301 on alveolar liquid clearance in ALI patients.

7. STUDY OBJECTIVES

7.1 Primary Objective

To assess the effect of orally inhaled AP301 on alveolar liquid clearance in ALI patients with the purpose to assess the treatment associated changes of extravascular lung water (EVLW) within 7 days of treatment.

7.2 Secondary Objectives

To assess:

- the treatment associated changes of oxygenation as measured by the PaO_2 / FiO_2 ratio until day 7 of therapy,
- the ventilatory plateau pressure until day 7 of therapy,
- the Murray lung injury score (a composite variable that includes components of oxygenation, compliance, positive end-expiratory pressure, and the appearance of the chest radiograph),
- ventilation parameters: tidal volume (V_t), positive endexpiratory pressure (PEEP), respiratory rate, FiO_2 , PIP, mean airway pressure, peak airway pressure until day 7 of treatment
- the duration of stay at ICU in the first 28 days,
- the duration of controlled ventilation period until extubation,
- the number of days free from ventilatory support [for > 48 h] in the first 28 days,
- the survival status at day 28,
- the local and systemic safety and tolerability and to identify possible dose related adverse events.

8. STUDY DESIGN

This will be an interventional, randomized, double-blind, placebo-controlled, parallel-group, monocentric, phase II “proof of concept” study. After patients meet the inclusion criteria and do not

display any exclusion criteria, they will be randomized to receive either orally delivered doses of 87.6 mg AP301 or a placebo solution every 12 hours via oral inhalation for 7 days.

To be eligible, subjects must be mechanically ventilated adult male and female patients at AKH, Vienna, Austria, with an onset of the ALI within the last 48 hours.

The treatment will last 7 days, after 28 days (+7) a follow-up interview via telephone will take place.

All study procedures will be performed at the Intensive Care Units (ICU).

<u>Study Flow:</u>

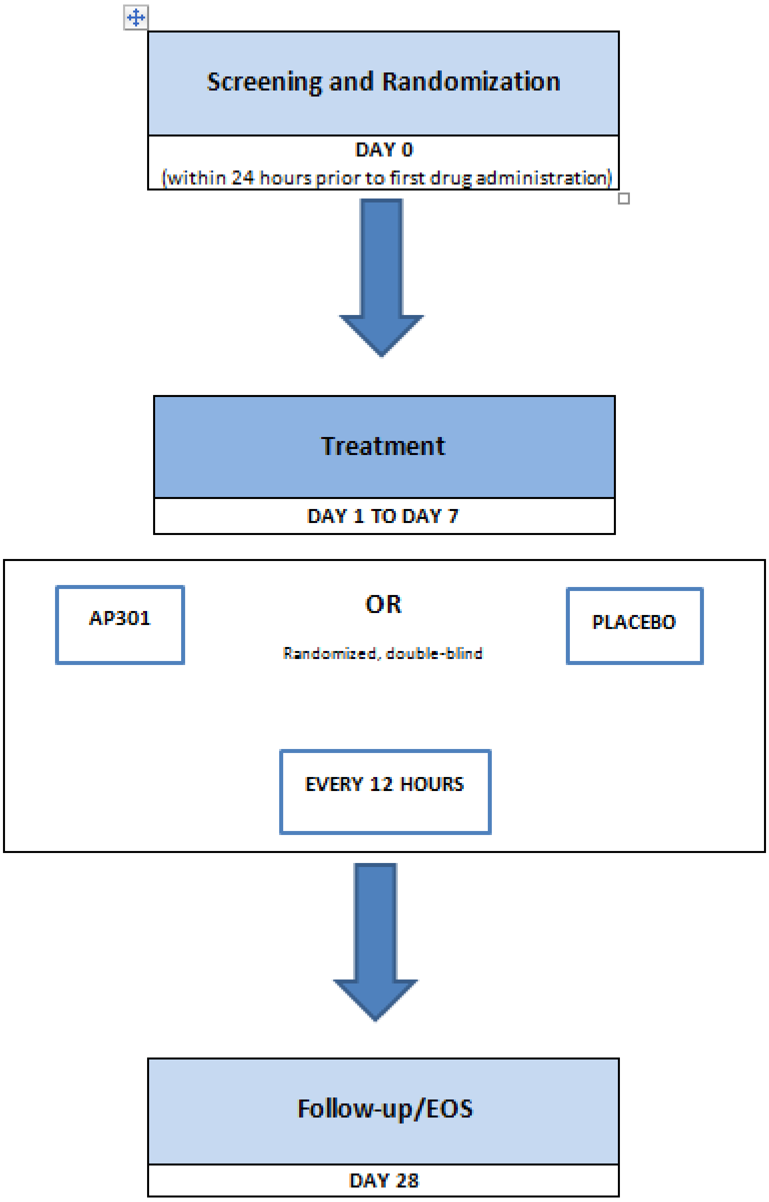

8.1 Study population

8.1.1 Subject population

There will be 2 parallel groups (Study group I / AP301 group, and study group II / Placebo group). It is planned to randomize a total of 40 patients in a 1:1 ratio to each of the treatment groups to target for about 20 evaluable patients / group (i.e. a total of 40 evaluable patients) available for evaluation. Subjects will be stratified regarding SOFA-Score (Sepsis-related Organ Failure Assessment) (Vincent, et al., 1996). Cut-off will be made at SOFA scores > 10. Therefore, there will be 2 subgroups (SOFA score ≤ 10, SOFA score ≥ 11). The GOCA score (Artigas, et al., 1998) will be assessed too, to be able to determine different causes of ALI.

8.1.2 Inclusion criteria

Intubated and mechanically ventilated male and female patients of the Intensive Care Units of the Department of Anaesthesiology of the Medical University of Vienna who:

- meet the criteria of ALI as defined by the American – European Consensus Conference on ALI / ARDS (Bernard, et al., 1994):
- Onset of ALI within 48 hours
- Bilateral infiltrates seen on frontal chest radiograph
- PCWP ≤ 18 mm Hg or no clinical evidence of left atrial hypertension
- paO_2 / FiO_2 ratio ≤ 300 mm Hg
- have EVLW in PICCO® at screening ≥ 8ml/PBW
- meet criteria for extensive hemodynamic monitoring according to investigators discretion
- are ≥ 18 years
- ICU Patients being mechanically ventilated and are stable in this condition for at least 8 hours
- Negative pregnancy test and adequate contraception in female patients of childbearing potential

Informed consent:

For patients that are temporarily unable to consent (e.g. comatose patients) a subsequent informed consent has to be provided according to Austrian Law (AMG §43a).

8.1.3 Exclusion criteria

Medical Issues

- History of clinically relevant allergies or idiosyncrasies to AP301 or any other inactive ingredient(s) of the investigational product
- Brainstem death at screening
- Current evidence of septic shock as defined by the Surviving Sepsis Campaign (Presence of acute organ dysfunction secondary to infection plus hypotension with systolic blood pressure < 90 mm Hg or mean arterial pressure (MAP) < 70 mm Hg for not less than one hour which cannot be reversed with fluid resuscitation)
- Neutrophil count < 0.3 x 10^9 L
- Patients under immunosuppression: high dose steroids (> 80 mg Prednisolone /d; > 300 mg hydrocortisone / d), currently undergoing cancer treatment (chemotherapy or biological) or therapy with other immunosuppressive agents for organ transplantation within 2 weeks
- BMI< 18.5 and >35
- Cardiogenic pulmonary edema diagnosed by echocardiography or pulmonary artery catheter
- Pregnancy / lactation or intention to fall pregnant during the time course of the study. Women of childbearing potential as well as men of procreative capacity who are not using adequate contraception.

General Issues

- Participation in other interventional drug trials

8.1.4 Females of childbearing age

Females can be included if they are unlikely to conceive, as indicated by at least one of the following points:

- Surgical sterilization
- Postmenopausal female > 45 years of age with > 2 years since last menses
- Non-sterilized, premenopausal, non-amenorrhoe female who agrees to abstain from heterosexual activity or to use an adequate method of contraception. Acceptable methods of

birth control are intrauterine device (IUD), diaphragma with spermicide, vasectomy, condom or hormonal contra-ceptives.

A pregnancy test will be performed at screening.

8.1.5 Study duration

The duration of treatment for each patient will be 7 days. On day 28 (+7) a follow-up and end of study interview will take place via telephone.

8.1.6 Withdrawal and replacement of subjects

Criteria for withdrawal

Subjects may prematurely discontinue from the study at any time. Premature discontinuation from the study is to be understood when the subject did not undergo End of Study (EOS) interview and / or all pivotal assessments during the study.

Subjects must be withdrawn under the following circumstances:

- at their own request
- if the investigator feels it would not be in the best interest of the subject to continue
- if the subject violates conditions laid out in the consent form / information sheet or disregards instructions by the study personal
- If the patient is in need of medication, which is not allowed according to 9.1.8
- allergies or idiosyncrasies to AP301 or any other inactive ingredient(s) of the investigational product

In all cases, the reason why subjects are withdrawn must be recorded in detail in the CRF and in the subject's medical records. Should the study be discontinued prematurely, all study materials (complete, partially completed and empty CRFs) will be retained.

Follow-up of patients withdrawn from the study

In case of premature discontinuation after study drug intake, the investigations scheduled for the EOS visit will be performed on the day of study drug discontinuation. The subjects will be advised that participation in these investigations is voluntary. Furthermore, they may request that from the time point of withdrawal no more data will be recorded and that all biological samples collected in the course of the study will be destroyed.

Replacement policy

A sample size of 20 evaluable patients / group will be achieved. If applicable, drop-outs will be included in the sample size by using the Last Observation Carried Forward-method to complete the primary efficacy variable EVWL. If a patient cannot be included, he/she will be replaced by using the next free number of the respective stratum list.

8.1.7 Premature termination of the study

The sponsor has the right to close this study at any time. The IEC and the competent regulatory authority must be informed within 15 days of early termination.

The trial will be terminated prematurely in the following cases:

- If adverse events occur which are so serious that the risk-benefit ratio is not acceptable.
- If the number of dropouts is so high that proper completion of the trial cannot realistically be expected.

9. METHODOLOGY

9.1 Study medication

Active agent and characteristics:

AP-301 is a TNF-α-derived alveolar epithelial sodium channel (ENaC) enhancing peptide that activates epithelial ion channels and facilitates fluid reabsorption in various animal models (in vitro, ex vivo, in situ and in vivo). In animal models AP 301 activates reabsorption of lung oedema of various pathophysiological origins, e.g. hydrostatic edema, the VALI- and ALI/ARDS-related permeability edemas, lung transplantation related edema, and edema caused by bacterial or viral pneumonias. The lectin-like domain of TNF-α also protects against the effects of free oxygen radicals and improves the epithelial cell function after tissue damage as seen in lung ischemia-reperfusion injury (LIRI).

The reference product will be a matching placebo formulation (0.9% NaCl solution (Ph.Eur.)).

The drug substance is the “Human Tumour Necrosis Factor alpha-derived peptide Cys-Gly-Gln-Arg-Glu-Thr-Pro-Glu-Gly-Ala-Glu-Ala-Lys-Pro-Trp-Tyr-Cys” and represents the “TIP-domain” of the Tumour Necrosis Factor alpha (TNF). The peptide is composed of 17 naturally occurring amino acids

and is circularised through an intramolecular disulfide bond between the N-terminal and C-terminal cysteine. It is soluble in water and has a molecular mass of 1,923.1.The drug substance AP301 peptide is produced by chemical peptide synthesis.

Manufacturer of Drug Product AP301 25 mg powder for reconstitution for solution for inhalation: Aptuit Glasgow, UK

Storage Instructions:

The drug product (Investigational Medicinal Product, IMP) "AP301 25 mg powder for reconstitution for solution for inhalation" is packed in Ph. Eur. Type I glass vials closed with a rubber stopper and sealed with a cap. It should be stored upright in a temperature monitored 2-8°C refrigerator until required. The refrigerator should be lockable.

The proposed shelf life at standard storage condition of 2-8°C is 15 months.

Route of administration:
Oral inhalation via nebulising device (Aeroneb® solo nebulisation system)

9.1.1 Dosage and administration

After patients meet inclusion criteria, in study group I orally delivered doses of 87.6 mg AP301 (dose per subject, 5 ml nebuliser filling dose) are inhaled every 12 hours (± 30 min), for a total of 7 days. 87,6 mg AP301 are based on 125 mg nebuliser filling dose.

This dose level is well below the highest dose level used in the phase I study. It represents a dose level corresponding to the most effective dose levels used in pharmacologic studies.

After patients meet inclusion criteria, in study group II Placebo solution (0.9% physiologic NaCl, / 5 ml nebuliser filling dose) is inhaled every 12 hours (± 30 min), for a total of 7 days.

To enable oral inhalation, reconstituted AP301 in WFI is converted into an inhalable aerosol by the Aeroneb Solo medicinal device. The Aeroneb Solo medicinal device is a product of Aerogen, Galway, Ireland and is a commercially available liquid nebuliser. The Aerogen Solo nebuliser has been approved in the European Community by CE-marking based on requirements of Annex II, Section 3.2. of Directive 93/42/EEC.

The nebulizer unit holds up to 6 ml of liquid medication. The nebulizer unit is clear to allow visual monitoring of medication levels and aerosolization. When the nebulizer unit is connected into the ventilator circuit, the silicon plug can be opened and closed in between doses without causing loss of circuit pressure.

9.1.2 Study drug interruption or discontinuation

The investigator must temporarily interrupt or permanently discontinue the study drug if continued administration of the study drug is believed to be contrary to the best interests of the patient.

The interruption or premature discontinuation of study drug might be triggered by an AE, a diagnostic or therapeutic procedure, an abnormal assessment (e.g., laboratory abnormalities), or for administrative reasons, in particular withdrawal of the patient's consent.

The reason for study drug interruption or premature permanent discontinuation must be documented in the CRF.

9.1.3 Study drug premature permanent discontinuation

Study drug premature permanent discontinuation due to an adverse event

If the reason for premature permanent discontinuation of study treatment is an AE, the patient should have a "Premature End of Study (EOS)" visit with all the assessments performed before the study drug discontinuation, whenever possible.

Study drug premature permanent discontinuation due to another reason than adverse event

If the reason for premature permanent discontinuation of study treatment is not an AE, the patient should be withdrawn from the study (withdrawal of consent) and have the end of study (EOS) visit with all the assessments performed before the study drug discontinuation, whenever possible.

9.1.4 Study drug packaging, labeling and delivery

The IMP "AP301 25 mg powder for reconstitution for solution for inhalation" will be packed to 10 vials into single cartons. Placebo solution (0.9% saline) will be packed to 10 vials into single cartons. Diluent (WFI) will be packed to 10 vials into single cartons. These cartons will be shipped under temperature control, depending on the recruitment status, to the "Anstaltsapotheke des Allgemeines Krankenhaus der Stadt Wien" (pharmacy). The pharmacy will be responsible for blinding.

At the pharmacy study medication will be provided ready for use and labeled according to Annex 13 of the Good Manufacturing Practice:

Prior to clinical use, AP301 will be reconstituted and labelled to maintain blinding. Briefly, 1 mL of Water for Injection (WFI) will be added to each vial of the IMP for reconstitution of the lyophilised AP301. The vial has to be tipped gently from side to side until the whole powder has dissolved. For

each orally delivered dose of 87.6 mg AP301 (dose per subject, corresponding to 5 ml nebuliser filling dose) 5 vials of the IMP AP301 have to be dissolved this way. The entire volume of these 5 vials (i.e. 5 ml) will be collected in a syringe. The closed and labelled syringe will be transported to the patient where the whole content of this syringe will be filled into the nebulizer reservoire chamber.

The initial nebuliser filling dose is 125 mg AP301 per 5 ml diluent. The reconstituted solution for inhalation or the placebo solution will be available in syringes. These syringes will be labelled according to the following label:

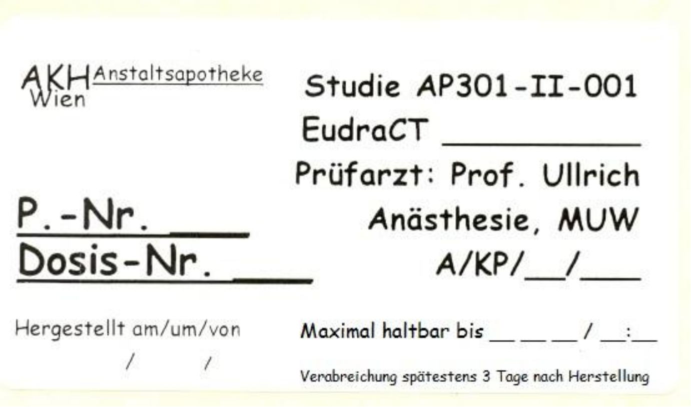
AKH Wien Anstaltsapotheke
Studie AP301-II-001
EudraCT ___________
Prüfarzt: Prof. Ullrich
Anästhesie, MUW
P.-Nr. ____
Dosis-Nr. ____
A/KP/__/___
Hergestellt am/um/von
/ /
Maximal haltbar bis __ __ __ / __:__
Verabreichung spätestens 3 Tage nach Herstellung

9.1.5 IMP administration & handling

Dosage and administration

The intended mode of administration is oral inhalation of the reconstituted solution or placebo.

In general the timepoints for inhalations should be scheduled around 08:00 a.m. (+/- 30 minutes) and 08:00 p.m. (+/- 30 minutes). The 12 hours sequence should be followed as closely as possible. If applicable to the routine of the ICU inhalation timepoints can be shifted to 10:00 a.m. (+/- 30 minutes) and 10 p.m (+/- 30 minutes).

If a patient is extubated for more than 48 hours the patient will not receive any other medication in case of re-intubation. If a patient is re-intubated within less than 48 hours administration of IMP will be continued until day 7.

Day 1:

The first administration will be performed as soon as possible, depending on the patient`s transfer to the ICU, if the interval to the next timepoint for inhalation is more than 8 hours. If the following administration of IMP is planned in less than 8 hours, the IMP shold be given at the normal timepoint (08.00 a.m. / 08.00 p.m.).

Day 2 to 7:

The exact timepoints of the administration schedule should be upheld.

Handling of IMP & nebuliser

The investigational medical product is available as solution. The investigational and reference products must be administered according to the procedures described herein. Only authorized site staff may dispense the study medication.

The nebuliser unit has a clear colour to allow visual monitoring of medication levels and aerosolisation. A compatible T-piece (which will be provided by the Sponsor) securely connects the nebuliser chamber into the breathing circuit. The T-piece connections are standard male and female 22mm conical ports and connect to standard patient breathing circuits. To ensure proper nebulisation, the nebuliser should be maintained in a vertical orientation. The silicon plug on top of the nebulizer chamber should be closed to guarantee a sufficient nebulization. For further information please refer to the standard operation proceredures (SOPs).
Detailed instructions for study drug preparation will also be given to the clinical team in advance before the study start.
Actual timing of each oral inhalation will be recorded in CRF.

9.1.6 Drug accountability

The Investigator will be responsible for the accountability (dispensing, inventory, and record keeping), exercising accepted medical and pharmaceutical practices, and adhering to all Good Clinical Practices (GCP) guidelines and local/national regulations.

Under no circumstances the Investigator will allow the study drug to be used other than as directed by this protocol. A “Drug Inventory Log” will be kept as an accurate and timely record of the receipt and dispensing of study drug to the subject.

Documentation should be kept regarding (a) documentation of receipt, (b) study drug dispensing log, (c) study drug accountability, and (d) all shipping service receipts. Authorized monitors will check and verify the drug accountability.

9.1.7 Procedures to assess subjects compliance

The study medication will only be administered by authorized study personnel.

9.1.8 Concomitant medication

The following concomitant medications are not allowed:

Immunosuppressives:

- high dose steroids (> 80 mg Prednisolone /d; > 300 mg hydrocortisone / d)
- cancer treatment (chemotherapy or biological)
- therapy with other immunosupressive agents for organ transplantation

9.2 Randomization and stratification

Subjects will be stratified regarding SOFA-Score (Sepsis-related Organ Failure Assessment) (Vincent, et al., 1996). Cut-off will be made at SOFA scores > 10. Therefore, there will be 2 subgroups (SOFA score ≤ 10, SOFA score ≥ 11). The GOCA score (Artigas, et al., 1998) will be assessed too, to be able to determine different causes of ALI.

Each subject will be randomised to one of 2 groups according to a randomization schedule prepared under the control of Bioconsult GmbH.

A stratified Randomization (strata: SOFA score ≤ 10, SOFA score ≥ 11) will be performed. Treatment allocation will be done using random permuted blocks within strata.

Randomization data will be kept strictly confidential, accessible only to authorised persons, until the time of unblinding. Authorised persons are:

- IT manager of Bioconsult GmbH,
- Drug packaging contractor.

9.3 Blinding

This is a double-blind study. Patients and all personnel involved with the study will be blinded. AP301 and saline solution will have similar appearance. All personnel directly involved with the study, including the Investigator, will be blinded to the medication codes. The following staff will be unblinded due to their profession: responsible staff for the preparation of code break envelopes, responsible persons of preparation of randomization listing, responsible persons at the pharmacy for labeling and preparation of IMP. The pharmacy will receive envelopes for every randomization number. These envelopes contain information about the affiliation of the randomization number (IMP or placebo). The pharmacy then prepares the IMP or placebo for use and labels the syringe to maintain blinding for study staff and patients.

9.3.1 Emergency procedure for unblinding

Unblinding of study patients is prohibited unless there is an emergency. Randomization lists will be maintained in a secure location by Bioconsult GmbH and at the pharmacy. With each medication a sealed envelope (Code Break Envelope) will be available. This envelope contains information on the type of study medication (IMP or placebo) and should be opened by the investigator only in case of a serious event. The instruction for notification of SAEs must be followed.

9.3.2 Unblinding at the end of the study

All data analyses will be performed by Bioconsult GmbH, Breitenfurt, after the study is completed and the database is released for unblinding.

9.4 Benefit and risk assessment

The standard battery of safety pharmacology studies did not reveal drug-related adverse effects in any of the animal models.

During the Phase I study no deaths or serious adverse events occurred. Only five possibly AP301 related adverse events (AEs) were found in four of 48 subjects, but all five were deemed mild by the Investigator and resolved spontaneously (see Table 2). No signs of local airway / lung intolerability were noted.

In the planned phase II study, seriously ill patients, already on ventilation, will receive the IMP on top of standard therapy directly over the ventilation system. These circumstances and the fact that examinations such as lung function measurements or blood samplings will be performed with the

patient under sedation, mean that procedure related AEs are unlikely to occur. Usually patients on the ICU have arterial catheters and blood draw is without risk. If the patient is conscious, he might feel a slight dragging pain. If venipuncture is necessary for blood draw, this might lead to a hematoma and might cause some pain.

AP301 was quantifiable in plasma only in extremely small fractions of the orally delivered dose for a brief time period, shortly after inhalation, thereby suggesting that systemic bioavailability is extremely low. Accordingly no significant systemic effects are expected.
This study is part of a clinical development program of AP301, which might be a treatment option for patients with life-threatening pulmonary oedema.

Therefore, the benefits of this trial outweigh the potential risks of participation.

9.5 Study procedures

9.5.1 General rules for trial procedures

- All study measures like blood sampling and measurements (vital parameters, ECG, etc.) have to be documented with date (dd:mm:yyyy).
- There is a specific sequence for study procedures that should be followed: EVLW must be measured within 60 to 120 minutes after inhalation of IMP or placebo. All parameters should be assessed, if possible, as standardized as possible (e.g. daily between 08:00 a.m. and 10:00 a.m.).
- The dates of all procedures should be according to the protocol. The time margins mentioned in the study flow chart are admissible. If for any reason, a study procedure is not performed within scheduled margins a protocol deviation should be noted, and the procedure should be performed as soon as possible or as adequate.
- If a patient is extubated for more than 48 hours the patient will not receive any other study medication in case of re-intubation. Only if a patient is re-intubated within less than 48 hours administration of IMP or placebo will be continued until day 7.

9.5.2 Screening investigation (Day 0)

Written informed consent must be obtained prior to embarking into any study related procedures. For patients that are temporarily unable to consent (e.g. comatose patients) a subsequent informed consent has to be provided.

The screening investigation aims to screen patients for potential participation. Inclusion and exclusion criteria will be checked.

Demographic data (age, ethnicity, height, weight and gender), vital signs (pulse rate, respiratory rate and temperature), the medical history (including the Glasgow Coma Scale) as well as concomitant medication will be documented. A blood sample for the evaluation of blood gases, clinical chemistry and hematology will be taken. The following scores will be obtained: GOCA (Artigas, et al., 1998), SOFA (Vincent, et al., 1996) and the lung injury score (Murray, Matthay, Luce, & Flick, 1988). The EVLW will be measured via PiCCO® technique. Hemodynamic parameters (HR, systolic BP, MAP, CI, PBV, 24 hours fluid balance and cardiac output) will be analyzed. For women with childbearing potential, a pregnancy test will be performed. Furthermore, the oxygenation index (PaO_2/FiO_2), the ventilatory plateau pressure and ventilation parameters (V_t, PEEP, PIP, respiratory rate, FiO_2, mean airway pressure and peak airway pressure) will be obtained.

9.5.3 Treatment Phase (Day 1 to 7)

Randomization will be performed prior to first administration of study medication.

All of the following parameters will be measured once in 24 hours, EVLW values will be assessed every 12 hours (between 60 and 120 minutes after administration of IMP) until day 7.

Vital signs will be measured (pulse rate and respiratory rate). A blood sample for the evaluation of blood gases, clinical chemistry and hematology will be taken. The lung injury score (Murray, Matthay, Luce, & Flick, 1988) will be obtained. The EVLW will be measured via PiCCO® technique. Hemodynamic parameters will be analyzed. Adverse events and the concomitant medication will be recorded. Furthermore, the oxygenation index, the ventilatory plateau pressure and ventilation parameters will be obtained.

In case that a patient will be extubated during the treatment phase, no more study medication will be administered. If the patient is reintubated within 48 hours after extubation, study medication will be administered again (until day 7). Nonetheless, all parameters that still will be measured after extubation will be documented.

9.5.4 End-of-study (EOS) interview (Day 28 + 7 days)

On day 28 (+ 7) a follow-up/ EOS interview will take place via telephone. Survival will be documented. Adverse events and concomitant medication will be documented and the ventilation-free days (for > 48h) until day 28 will be listed. Furthermore, the duration of stay at ICU in the first 28 days and the duration of controlled ventilation period until extubation will be recorded.

9.5.5 Early termination (ET)

Patients who are withdrawn from the study prematurely by the investigator or the sponsor, or who withdraw consent themselves, will undergo all investigations as described for any treatment day respectively for the end-of-study interview. The reason(s) for ET must be specified and documented in the source data and on the appropriate page of the eCRF.

9.5.6 Methods of evaluation

Vital signs

Vital signs (including, pulse rate and respiratory rate) will be assessed at screening visit and daily until end of treatment. The temperature will be measured only at screening.

Demography

Age, gender, ethnicity, height and weight will be recorded at screening visit.

Body weight

The weight will be assessed at screening visit and the BMI will be calculated. The BMI has to be > 18.5 and < 35 (refer to inclusion criteria).

Medical history

Medical History will be documented at screening. Significant findings prior to the start of study drug will be recorded on the relevant Medical History page of the CRF. Clinically relevant findings after or during study drug application and meeting the definition of an AE must be reported on the corresponding AE page in the CRF. The value of the Glasgow Coma Scale will be assessed retrospectively. This is a scale to assess the conscious state of a patient.

Concomitant medication

Concomitant medication and changes in concomitant medication will be documented at screening as well as daily during treatment and on day 28 (+7).

Laboratory assessments

Clinical laboratory tests will be performed at screening visit as well as daily during treatment.

The clinical laboratory tests to be performed:

Category	Parameters
Hematology	WBC, haematocrit, haemoglobin, platelet count
Chemistry	Creatinine, sodium, potassium, BUN, bilirubin
Blood gases	Arterial pH, PaO_2, FiO_2, biocarbonate, sO_2, $paCO_2$
Other	

PiCCO®: EVLW

The extravascular lung water will be assessed at screening via PiCCO® technique. It will be indexed to predicted body weight (PBW).

The predicted body weight is calculated as follows:

PBW for women: 45.5 + 0.91 x [height(cm) – 152.4]

PBW for men: 50 + 0.91 x [height(cm) – 152.4]

PiCCO® allows continuous hemodynamic monitoring. It works via a central venous and an arterial access. Furthermore, the changes of extravascular lung water will be assessed every 12 hours (within 60 to 120 minutes after inhalation of IMP or placebo) until end of treatment.

GOCA (Artigas, et al., 1998)

GOCA (Gas exchange, Organ failure, Cause, and Associated conditions) is a scoring system developed for patients with ARDS. It assesses general health and the severity of lung injury. The GOCA score will be assessed at screening.

Murray Lung Injury Score (Murray, Matthay, Luce, & Flick, 1988)

The Murray Lung Injury Score is a composite variable that includes components of oxygenation, lung compliance, positive end-expiratory pressure and the appearance of the chest radiograph. It evaluates the extent of acute pulmonary damage. The Lung Injury Score will be assessed at screening as well as daily until end of treatment.

SOFA (Vincent, et al., 1996)

The Sepsis-related Organ Failure Assessment score is a scoring system to evaluate the condition of patients at ICUs. It considers different systems, such as respiratory or renal systems. The SOFA score will be assessed at screening as well as daily until end of treatment. Patients will be stratified according to this score.

Ventilation parameters

The ventilatory plateau pressure, tidal volume (V_t), positive endexpiratory pressure (PEEP), peak inspiratory pressure (PIP), respiratory rate, FiO_2, mean airway pressure and the peak airways pressure will be assessed at screening and daily until end of treatment.

Oxygenation index

The oxygenation index will be assessed at screening and furthermore daily the changes of oxygenation index until end of treatment. It will be calculated from PaO2 and FiO2 as the PaO2/FiO2 ratio (ratio of arterial oxygen tension to the fraction of inspired oxygen).

Hemodynamic parameters

The heart rate, systolic blood pressure, mean arterial pressure, PBV, 24 hours fluid balance, cardiac index and cardiac output (as measured by PiCCO®) will be assessed at screening and daily until end of treatment.

Pregnancy test

Pregnancy tests must be performed at screening for all women of child-bearing potential even if they are practicing active contraception. The pregnancy test will be performed by using kits reacting on elevated βHCG levels in urine. If patients are anuric βHCG levels in blood will be assessed.

10. SAFETY DEFINITIONS AND REPORTING REQUIREMENTS

10.1 Averse events (AEs)

10.1.1 Summary of known and potential risks of the study drug

The standard battery of safety pharmacology studies did not reveal drug-related adverse effects in any of the animal models.

So far AP301 has been investigated in Phase I. AP301 was generally well tolerated. The occurred AEs did not affect either the clinical conditions or the life style of subjects. No deaths occurred during the study. No Serious Adverse Events (SAE) or unexpected AEs (SUSARs) occurred throughout the study.

The table below summarises all AEs, which were judged to be possibly related to treatment, in all subjects.

Table 2 Number of subjects for each possibly related adverse event

System Organ Class [1]<br>Preferred Term [1]	D1<br>N=6<br>n	D2<br>N=6<br>n	D3<br>N=6<br>n	D4<br>N=6<br>n	D5<br>N=6<br>n	D6<br>N=6<br>n	P<br>N=12<br>n	Overall<br>N=48<br>n (%)
**Number of subjects for each related adverse event**	0	**1**	**1**	**1**	**1**	0	**1**	5 (10.4)
**Gastrointestinal disorders**	0	**1**	0	0	0	0	0	1 (2.1)
Abdominal pain	0	**1**	0	0	0	0	0	1 (2.1)
Flatulence	0	**1**	0	0	0	0	0	1 (2.1)
**Investigations**	0	0	0	0	**1**	0	0	1 (2.1)
WBC count decreased	0	0	0	0	**1**	0	0	1 (2.1)
**Nervous system disorders**	0	0	0	**1**	0	0	0	1 (2.1)
Headache	0	0	0	**1**	0	0	0	1 (2.1)
**Respiratory, thoracic and mediastinal disorders**	0	0	**1**	0	0	0	0	1 (2.1)
Diaphragmatic disorder	0	0	**1**	0	0	0	0	1 (2.1)

**Skin and subcutaneous tissue disorders**	0	0	0	0	0	0	**1**	1 (2.1)
Pruritus	0	0	0	0	0	0	**1**	1 (2.1)

D1: AP301 4.32 mg/Subjects D2: AP301 12 mg/Subjects D3: AP301 30 mg/Subjects
D4: AP301 60 mg/Subjects D5: AP301 90 mg/Subjects D6: AP301 120 mg/Subjects
P: Matching placebo formulation to AP301
1 MedDRA version 13.1

10.1.2 Definition of adverse events

An AE is any untoward adverse change from the subject's baseline condition, i.e., any unfavorable and unintended sign including an abnormal laboratory finding, symptom or disease which is considered to be clinically relevant by the physician that occurs during the course of the study, whether or not considered related to the study drug.

Adverse events include:

- Exacerbation of a pre-existing disease.
- Increase in frequency or intensity of a pre-existing episodic disease or medical condition.
- Disease or medical condition detected or diagnosed after study drug administration even though it may have been present prior to the start of the study.
- Continuous persistent disease or symptoms present at baseline that worsen following the start of the study.
- Lack of efficacy in the acute treatment of a life-threatening disease.
- Events considered by the investigator to be related to study-mandated procedures.
- Abnormal assessments, e.g., ECG and physical examination findings, must be reported as AEs if they represent a clinically significant finding that was not present at baseline or worsened during the course of the study.
- If laboratory test abnormalities are evaluated as an AE remains to the discretion of the investigator.

Adverse events do not include:

- Pre-planned interventions or occurrence of endpoints specified in the study protocol are not considered AE´s, if not defined otherwise (eg.as a result of overdose)
- Medical or surgical procedure, e.g., surgery, endoscopy, tooth extraction, transfusion. However, the event leading to the procedure is an AE. If this event is serious, the procedure must be described in the SAE narrative.

- Pre-existing disease or medical condition that does not worsen.
- Situations in which an adverse change did not occur, e.g., hospitalizations for cosmetic elective surgery or for social and/or convenience reasons.
- Overdose of either study drug or concomitant medication without any signs or symptoms. However, overdose must be mentioned in the Study Drug Log.

10.2 Serious Adverse Events (SAEs)

A Serious Adverse Event (SAE) is defined by the International Conference on Harmonization (ICH) guidelines and WHO GCP guidelines as any AE fulfilling at least one of the following criteria:

- Results in deaths.
- Life-threatening – defined as an event in which the subject was, in the judgment of the investigator, at risk of death at the time of the event; it does not refer to an event that hypothetically might have caused death had it been more severe.
- Requiring subject's hospitalization or prolongation of existing hospitalization – inpatient hospitalization refers to any inpatient admission, regardless of length of stay.
- Resulting in persistent or significant disability or incapacity (i.e., a substantial disruption of a person's ability to conduct normal life functions).
- Congenital anomaly or birth defect.
- Is medically significant or requires intervention to prevent at least one of the outcomes listed above.

Life-threatening refers to an event in which the subject was at risk of death at the time of the event. It does not refer to an event that hypothetically might have caused death if it were more severe.

Important medical events that may not immediately result in death, be life-threatening, or require hospitalization may be considered as SAEs when, based upon appropriate medical judgment, they may jeopardize the subject and may require medical or surgical intervention to prevent one of the outcomes listed in the definitions above.

10.2.1 Hospitalization – Prolongation of existing hospitalization

Hospitalization is defined as an overnight stay in a hospital unit and/or emergency room.

An additional overnight stay defines a prolongation of existing hospitalization.

The following is not considered an SAE and should be reported as an AE only:

- Treatment on an emergency or outsubject basis for an event not fulfilling the definition of seriousness given above and not resulting in hospitalization.

The following reasons for hospitalizations are not considered AEs, and therefore not SAEs:

- Hospitalizations for cosmetic elective surgery, social and/or convenience reasons.
- Standard monitoring of a pre-existing disease or medical condition that did not worsen, e.g., hospitalization for coronary angiography in a subject with stable angina pectoris.
- Elective treatment of a pre-existing disease or medical condition that did not worsen, e.g., hospitalization for chemotherapy for cancer, elective hip replacement for arthritis.

10.2.2 SAEs related to study-mandated procedures

Such SAEs are defined as SAEs that appear to have a reasonable possibility of causal relationship (i.e., a relationship cannot be ruled out) to study-mandated procedures (excluding administration of study drug) such as discontinuation of subject's previous treatment during a washout period, or complication of a mandated invasive procedure (e.g., blood sampling, heart catheterization), or car accident on the way to the hospital for a study visit, etc.

10.2.3 Suspected unexpected serious adverse reactions (SUSARs)

SUSARs are all serious adverse reactions with suspect causal relationship to the study drug that is unexpected (not previously described in the SmPC - Summary of Product Characteristics or Investigator’s brochure) and serious.

10.2.4 Pregnancy

Any pregnancy that occurs during study participation must be reported to the investigator/sponsor. To ensure subject safety, each pregnancy must be reported to the investigator/sponsor immediately. The pregnancy must be followed up to determine outcome (including premature termination) and status of mother and child. Pregnancy complications and elective terminations for medical reasons must be reported as an AE or SAE. Spontaneous abortions must be reported as an SAE.

Any SAE occurring in association with a pregnancy brought to the investigator’s attention after the subject has completed the study and considered by the investigator as possibly related to the investigational product, must be promptly reported to the principal investigator/sponsor.

In addition, the investigator must attempt to collect pregnancy information on any female partners of male study subjects who become pregnant while the subject is enrolled in the study. Pregnancy information must be reported to the investigator/sponsor as described above.

10.3 Severity of adverse events

The severity of clinical AEs is graded on a three-point scale: mild, moderate, severe, and reported on specific AE pages of the CRF.
If the severity of an AE worsens during study drug administration, only the worst intensity should be reported on the AE page. If the AE lessens in intensity, no change in the severity is required.
If an AE occurs during a washout or placebo run-in phase and afterwards worsens during the treatment phase, a new AE page must be filled in with the intensity observed during study drug administration.

Mild
Event may be noticeable to subject; does not influence daily activities; the AE resolves spontaneously or may require minimal therapeutic intervention;

Moderate
Event may make subject uncomfortable; performance of daily activities may be influenced; intervention may be needed; the AE produces no sequelae.

Severe
Event may cause noticeable discomfort; usually interferes with daily activities; subject may not be able to continue in the study; the AE produces sequelae, which require prolonged therapeutic intervention.
A mild, moderate or severe AE may or may not be serious. These terms are used to describe the intensity of a specific event (as in mild, moderate, or severe myocardial infarction). However, a severe event may be of relatively minor medical significance (such as severe headache) and is not necessarily serious. For example, nausea lasting several hours may be rated as severe, but may not be clinically serious. Fever of 39°C that is not considered severe may become serious if it prolongs hospital discharge by a day. Seriousness rather than severity serves as a guide for defining regulatory reporting obligations.

10.4 Relationship to study drug

For all AEs, the investigator will assess the causal relationship between the study drug and the AE using his/her clinical expertise and judgment according to the following algorithm that best fits the circumstances of the AE:

1	**NOT RELATED**	This category applies to those adverse events which, after careful consideration, are clearly and incontrovertibly due to extraneous cause (disease, environment, etc.).
2	**UNLIKELY**	A clinical event, including laboratory test abnormality, with a temporal relationship to drug administration that makes a causal relationship improbable, and in which other drugs, chemicals, or underlying disease provide plausible explanations.
3	**POSSIBLY**	A clinical event, including laboratory test abnormality, with a reasonable time sequence to administration of the drug, but which could also be explained by concurrent disease or other drugs or chemicals. Information on drug withdrawal may be lacking or unclear.
4	**PROBABLY**	A clinical event, including laboratory test abnormality, with a reasonable time sequence to administration of the drug, unlikely to be attributed to concurrent disease or other drugs or chemicals, and which follows a clinically reasonable response on withdrawal (dechallenge). Rechallenge information is not required to fulfil this definition.
5	**DEFINITELY**	A clinical event, including laboratory test abnormality, occurring in a plausible time relationship to drug administration, and which cannot be explained by concurrent disease or other drugs or chemicals. The response to withdrawal of the drug (dechallenge) should be clinically plausible. The event must be definitive pharmacologically or phenomenologically, using a satisfactory rechallenge procedure if necessary.

10.5 Reporting procedures

A special section is designated to adverse events in the case report form. The following details must thereby be entered:

- Type of adverse event

- Start (date and time)
- End (date and time)
- Severity (mild, moderate, severe)
- Serious (no / yes)
- Unexpected (no / yes)
- Outcome (resolved, ongoing, ongoing – improved, ongoing – worsening)
- Relation to study drug (not related, unlikely, possibly, probably, definitely))

Adverse events are to be documented in the case report form in accordance with the above mentioned criteria.

10.5.1 Reporting procedures for SAEs

All unexpected SAEs must be reported to the KKS by FAX or email within 24 hours of learning of the occurrence using the SAE Case Report Form.

Please fax to:

Koordinierungszentrum Klinische Studien
Medical University of Vienna
Borschkegasse 8b
A-1090 Vienna
Facsimile: +43-1-40400-2372
E-mail: johannes.pleiner@meduniwien.ac.at
Tel: +436601120300

All the information requested on the SAE Form should be provided as available. The SAE form must be filled out and signed by the Investigator. All of those events must also be recorded in the appropriate CRF page(s).

The following details should at least be available:

- Patient initials and number
- Patient: date of birth, sex, ethical origin
- The suspected investigational medical product (IMP)
- The adverse event assessed as serious
- Short description of the event and outcome

If applicable, the initial report should be followed by the Follow up report, indicating the outcome of the SAE.

The Safety Department will review all incoming SAE reports for accuracy and completeness. SAEs will be reported to the appropriate authorities and Ethics Committees according to country-specific regulations i.e. summarized at regular intervals of at least one year.

10.5.2 Reporting procedures for SUSARs

It must be remembered that the regulatory authorities, and in case of SUSARs which could possibly concern the safety of the study participants, also the Institutional Review Board / Independent Ethics Committee (IRB / IEC) are to be informed. Such reports shall be made by the study management and the following details should be at least available:

- Patient initials and number
- Patient: date of birth, sex, ethical origin
- Name of investigator and investigating site
- Period of administration
- The suspected investigational medical product (IMP)
- The adverse event assessed as serious and unexpected, and for which there is a reasonable suspected causal relationship to the IMP
- Concomitant disease and medication
- Short description of the event:
    - Description
    - Onset and if applicable, end
    - Therapeutic intervention
    - Causal relationship
    - Hospitalization of prolongation of hospitalization
    - Death, life-threatening, persistent or significant disability or incapacity

Electronic reporting should be the expected method for reporting of SUSARs to the competent authority. In that case, the format and content as defined by Guidance (28) should be adhered to. The latest version of MedDRA should be applied. Lower level terms (LLT) should be used.

10.5.3 Annual Safety Report

The Annual Safety Report will be provided by the sponsor at least once a year.

This report will also be presented annually to the Independent Ethics Committee (IEC) and to the competent authorities.

10.5.4 Safety Monitoring Board

A Safety Monitoring Board will be installed for the case of safety concerns and the need of decisions regarding the study progress made by the Sponsor.

11. FOLLOW-UP

11.1 Follow-up of study participants including follow-up of adverse events

A follow-up/ end of study interview will be performed on day 28 (+7), about 21 days after end of treatment.
Follow-up of all SAEs will be done until the event is resolved or the event or sequel is stabilized.

Any medical-related issues or questions requiring immediate resolution or action should be directed to the Medical Supervisor.

11.2 Treatment after end of study

No special treatment after the end of the study is required.

12. STATISTICAL METHODOLOGY AND ANALYSIS

All data analyses will be performed by Bioconsult GmbH, Breitenfurt, after the study is completed and the database is released for unblinding. Statistical programming and analyses will be performed using SAS® Version 9.2 and SPSS® Version 19.
The statistical analyses described in this section will be performed as further outlined in the Statistical Analysis Plan (SAP), which will be finalized prior to unblinding of the database. All data will be listed by treatment group for subjects in the enrolled population, unless otherwise specified.
Continuous data will be summarized by treatment group using the following descriptive statistics: number, mean, standard deviation (SD), minimum, lower quartile (if appropriate), median, upper quartile (if appropriate) and maximum. Categorical data will be summarized by treatment group as the number and percentage of subjects in each category.

12.1 Analysis sets

Two different analysis sets are defined

- (Modified) Intention to treat set (ITT)

  This analysis set includes subjects who were randomized (and received at least one dose study drug).

- Per-protocol set (PP)

  This analysis set comprises all subjects who received study drug (at least one dose) and did not violate the protocol in a way that might affect the evaluation of the effect of the study drug(s) on the primary objective, i.e., without major protocol violations.

After data base close and before unblinding, an "unblinding session" will be held to define the study populations.

12.2 Sample size considerations

This study is intended to show the superiority of AP301 vs. Placebo, using "EVLW" (AUC between Day 0 and day 7) as Primary Efficacy Variable.

A mean difference of at least 40% between the two application conditions (AP301 vs. Placebo) will be supposed for EVLW (baseline SD about 40 %), indicating statistically significant reduction of EVLW by use of AP301 compared to Placebo.

A sample size of 40 (20 / group) will be sufficient to show a statistically significant difference between Verum and Placebo with $p < 0.05$ (two sided) and a power of 80%.

12.3 Relevant protocol deviations

There are to be no deviations from this protocol. Any subject whose treatment deviates from the protocol or who is not qualified for the study, may be ineligible for analysis and may compromise the study. Subjects not enrolled in the study cannot receive study drug.

The Investigator and research team must comply with all applicable national and local laws. All protocol deviations will be listed in the study report.

12.4 Statistical analysis plan

The statistical analyses described in this section will be performed as further outlined in the Statistical Analysis Plan (SAP), which will be finalised prior to unblinding of the database.

12.5 Missing, unused and spurious data

A sample size of 20 evaluable patients / group will be achieved. If applicable, drop-outs will be included in the sample size by using the Last Observation Carried Forward-method with respect to the primary efficacy variable "EVWL". If a patient cannot be included, he/she will be replaced by using the next free number of the respective stratum list.

12.6 Endpoints analysis

All efficacy data will be listed by treatment group and subject for the full analysis population. Subjects in the PP population will be identified with an asterisk.

12.6.1 Primary endpoint analysis

The primary efficacy variable will be summarised for subjects in both the full analysis and PP population. The full analysis will be the primary analysis population. All other efficacy variables will be summarised for subjects in the full analysis population only.
The primary efficacy variable will be EVWL (measured with PiCCO® technique) between day 0 and day 7 of treatment.

The hypothesis to be tested is superiority regarding the change of the primary efficacy variable "EVWL" in the "AP301 group" in comparison to the "Placebo group".

The null hypothesis is defined as
H_0: Mean EVWL (AUC between Day 0 and Day 7) in the AP301 group = Mean EVWL (AUC between Day 0 and Day 7) in the Placebo group.

The alternative hypothesis is defined formal as
H_1: Mean EVWL (AUC between Day 0 and Day 7) in the AP301 group < Mean EVWL (AUC between Day 0 and Day 7) in the Placebo group.

Statistical tests will be two-tailed at the 5% significance level (Power=80%). The primary efficacy variable (EVWL) will be analyzed confirmatory between the two conditions (AP301 vs. Placebo) using an ANCOVA (analysis of covariance) model or the non parametric Mann-Whitney U-test, dependant on the distribution of data.

12.6.2 Secondary endpoint analysis

Secondary efficacy variables are:

1. the treatment associated changes of oxygenation as measured by the PaO_2 / FiO_2 ratio until day 7 of therapy,
2. the ventilatory plateau pressure until day 7 of therapy,
3. the Murray lung injury score (a composite variable that includes components of oxygenation, compliance, positive end-expiratory pressure, and the appearance of the chest radiograph),
4. ventilation parameters: tidal volume (V_t), positive endexpiratory pressure (PEEP), respiratory rate, FiO_2 , PIP, mean airway pressure, peak airway pressure until day 7 of treatment
5. the duration of stay at ICU in the first 28 days,
6. the duration of controlled ventilation period until extubation,
7. the number of days free from ventilatory support [for > 48 h] in the first 28 days,
8. the survival status at day 28,
9. the local and systemic safety and tolerability and to identify possible dose related adverse events.

Secondary efficacy variables will be presented using appropriate descriptive methods, and will be analysed in an explorative sense: Mann-Whitney U-Test (Variable 1, 2, 3, 4), Kaplan-Meier estimates (variable 5, 6, 7, 8), and contingency tables (variable 9)

Statistical tests of the secondary efficacy variables and the p-values attached to them will be regarded as descriptive and not as tests of hypotheses.

12.6.3 Safety and tolerability endpoints

All safety data will be listed for subjects in the safety population.

AEs will be classified into standardised terminology from the verbatim description (Investigator term) according to the MedDRA Coding Dictionary. AEs will be classified by MedDRA term and associated system organ class.

Although a MedDRA term may be reported more than once for a subject, that subject will be counted only once in the incidence count for that MedDRA term.

The number and percentage of subjects reporting AEs will be summarised by treatment group, system organ class, MedDRA term, and phase.

12.6.4 Baseline parameters and concomitant medications

Laboratory Values:

Clinical laboratory values will be evaluated for each laboratory parameter by subject. Abnormal laboratory values will be identified as those outside (above or below) the normal range. Reference (normal) ranges for laboratory parameters will be included in the clinical study report for this protocol.

All clinical laboratory results will be evaluated for clinically notable abnormalities.

Vital Signs:

Vital sign values will be evaluated on a regular basis by subject.

Concomitant medication will be listed using ATC Codes.

12.7 Interim analysis

Not applicable.

12.8 Software program(s)

Statistical programming and analyses will be performed using SAS® Version 9.2 and SPSS® Version 19.

13. DOCUMENTATION AND DATA MANAGEMENT

13.1 Documentation of study results

A subject screening and enrollment Log will be completed for all eligible or non-eligible subjects with the reasons for exclusion.

13.1.1 Case report form (CRF)

An “eCRF“ will be used for this study. For each subject enrolled, regardless of study drug initiation, an eCRF must be completed. This also applies to those subjects who fail to complete the study. If a subject withdraws from the study, the reason must be noted on the eCRF. Case report forms are to be completed on an ongoing basis.
eCRF entries and corrections will only be performed by study site staff, authorized by the investigator.
In an “eCRF” all forms should be completed and must be legible. Changes will be saved (audit trail). The entries will be checked by trained personnel (Monitor) and any errors or inconsistencies will be checked immediately.
Datamanagement for the eCRFs of this study will be performed by Softwaremanufaktur Grünberg und Redl OG. The statistical analyses will then be performed by Bioconsult GmbH.

13.1.2 Data Collection

The collected data are entered into an interactive form (eCRF). The eCRFs will be compared to source documents and verified following guidelines established before study onset as detailed in the Monitoring Plan. Maintenance of the study database will be performed by Softwaremanufaktur Grünberg & Redl OG.

13.1.3 Identification data to be considered as source data

The following documents are considered to be source documents beside the general patient files:

Informed consent form
Drug dispensing/inventory Log
Subject identification List
Subject screening and enrolment Log

13.2 Safekeeping

The investigator will maintain adequate and accurate records to enable the conduct of the study to be fully documented and the study data to be subsequently verified (according to ICH-GCP “essential documents”). These documents will be classified into two different categories: investigator's file, and subject clinical source documents.

The investigator's file will contain the protocol/amendments, EudraCT forms, eCRF printouts, standard operation procedures (SOPs), data clarification and query forms, EC/IRB and Health Authority approval with correspondence, informed consent, drug records, staff curriculum vitae and authorization forms, screening and enrollment logs, and other appropriate documents/correspondence as per ICH/Good Clinical Practice (GCP) and local regulations.
Subject clinical source documents include, but are not limited to subject hospital/clinic records, physician's and nurse's notes, appointment book, original laboratory reports, ECG, X-ray, pathology and special assessment reports, consultant letters, etc.

These two categories of documents must be kept on file by the investigator for as long as needed to comply with national and international regulations (in Austria 15 years after discontinuing clinical development or after the last marketing approval). If source documents are not durable as long as needed (e.g. ECG printouts) they must be preserved as a copy. No study document should be destroyed without prior written approval from the Sponsor.
When source documents are required for the continued care of the subject, appropriate copies should be made for storing outside of the site.

13.3 Quality Control and Quality Assurance

13.3.1 Periodic Monitoring

The designated monitor will contact and visit the investigator regularly and will be allowed to have access to all source documents needed to verify the entries in the CRFs and other protocol-related documents provided that subject confidentiality is maintained in agreement with local regulations. It will be the monitor's responsibility to inspect the CRFs at regular intervals according to the monitoring plan throughout the study, to verify the adherence to the protocol and the completeness, consistency and accuracy of the data being entered on them. The monitoring standards require full verification for the presence of informed consent, adherence to the inclusion/exclusion criteria, documentation of SAEs and the recording of the main efficacy, safety, and tolerability endpoints.
Monitoring will be performed by the KKS (Koordinierungszentrum für klinische Studien, Med. Univ., Vienna). The monitor will be working according to SOPs of KKS and will provide a monitoring report after each visit for the Sponsor. 15 routine monitoring visits are planned beside one initiation visit and one close-out visit. Depending on the quality of the data, additional monitoring visits may be necessary according to the sponsor's discretion. 100% of source data will be checked by the monitor (100% SDV). The investigator will resolve discrepancies of data.

13.3.2 Audit and Inspections

Upon request, the investigator will make all study-related source data and records available to a qualified quality assurance auditor mandated by the sponsor or to competent authority inspectors. The main purposes of an audit or inspection are to confirm that the rights and welfare of the subjects have been adequately protected, and that all data relevant for assessment of safety and efficacy of the investigational product have appropriately been reported to the sponsor.

13.4 Reporting and Publication

13.4.1 Publication of study results

The findings of this study will be published by the investigator (author) and the sponsor (co-author) in a scientific journal and presented at scientific meetings. The manuscript will be circulated to all co-investigators before submission. Confidentiality of subjects in reports/publications will be guaranteed.

14. ETHICAL AND LEGAL ASPECTS

14.1 Informed consent of subjects

In this study it is expected that the vast majority of patients to be enrolled are temporarily unable to consent. For these patients (e.g. comatose patients), a subsequent informed consent has to be provided according to local law/ requirements.

In case a patient is able to consent, the patient must give written consent to participation in the study prior to embarking into any study related procedures, after they got comprehensive instructions regarding the nature, significance, impact and risks of this clinical trial. During the instruction regarding the clinical trial all trial participants are to be made aware of the fact that they can withdraw their consent – without giving reasons – at any time without their further medical care being influenced in any way.

In addition to the comprehensive instructions given to the trial participants by the investigator, the trial participants also receive a (if applicable: subsequent) written patient information sheet in comprehensible language, explaining the nature and purpose of the study and its progress.

The patients must agree to the possibility of study-related data being passed on to relevant authorities.The patients must be informed in detail of their obligations in relation to the trial participants insurance in order not to jeopardize insurance cover.

14.2 Acknowledgement / approval of the study

The designated CRO will submit this protocol and any related document provided to the subject (such as subject information used to obtain informed consent) to an Ethics Committee (EC) or Institutional Review Board (IRB). Approval from the committee must be obtained before starting the study.

The clinical trial shall be performed in full compliance with the legal regulations according to the Austrian Drug Law (AMG - Arzneimittelgesetz).

An application must also be submitted to the Austrian Competent Authorities (Medizinmarktaufsicht / Bundesamt für Sicherheit im Gesundheitswesen (BASG)) and registered to the European Clinical Trial Database (EudraCT) using the required forms. The timelines for (silent) approval set by national law must be followed before starting the study.

14.2.1 Changes in the Conduct of the Study

Protocol amendments

Proposed amendments must be submitted to the appropriate CA and ECs. Substantial amendments may be implemented only after CA/EC approval has been obtained. Amendments that are intended to eliminate an apparent immediate hazard to subjects may be implemented prior to receiving CA/EC approval. However, in this case, approval must be obtained as soon as possible after implementation.

Study Termination

If the sponsor or the investigator decides to terminate the study before it is completed, they will notify each other in writing stating the reasons of early termination. In terminating the study, the sponsor and the investigator will ensure the adequate consideration is given to the protection of the subject interests. The investigator, sponsor (or designated CRO on behalf of the sponsor) will notify the relevant CA and EC. Documentation will be filed in the Trial Master and Investigator Files.

Clinical Study Report (CSR)

Within one year after the final completion of the study, a full CSR will be prepared by the KKS and the statistician and submitted to the EC and the competent authority.

The Sponsor will be asked to review and sign the final study report.

14.3 Insurance

During their participation in the clinical trial the patients will be insured as defined by legal requirements. The investigator of the clinical trial will receive a copy of the insurance conditions of the 'patients insurance'. The sponsor is providing insurance in order to indemnify (legal and financial coverage) the investigator/center against claims arising from the study, except for claims that arise from malpractice and/or negligence. The compensation of the subject in the event of study-related injuries will comply with the applicable regulations.

Details on the existing patients insurance are given in the patient information sheet:

ZÜRICH Versicherungs-AG, Schwarzenbergplatz 15, 1010 Wien

Policy No.: 07229622-2

14.4 Confidentiality

The information contained in this document, especially unpublished data, is the property of the Sponsor. It is therefore provided to you in confidence as an investigator, potential investigator, or consultant, for review by you, your staff, and an Ethics Committee or Institutional Review Board. It is understood that this information will not be disclosed to others without written authorization from the Sponsor.

14.5 Ethics and Good Clinical Practice (GCP)

The investigator will ensure that this study is conducted in full conformance with the principles of the "Declaration of Helsinki" (as amended at the 56th WMA General Assembly, Tokyo, Japan, 2008) and with the laws and regulations of the country in which the clinical research is conducted.

The investigator of the clinical trial shall guarantee that only appropriately trained personnel will be involved in the study. All studies must follow the ICH GCP Guidelines (June 1996) and, if applicable,

the Code of Federal Regulations (USA). In other countries in which GCP Guidelines exist, the investigators will strictly ensure adherence to the stated provisions.

Therefore this study follows the EU Directive embedded in the Austrian drug act.

15. REFERENCES

Artigas, A., Bernard, G. R., Carlet, J., Dreyfuss, D., Gattinoni, L., Hudson, L., et al. (1998). The American-European Consensus Conference on ARDS, part 2: ventilatory, pharmacologic, supportive therapy, study design strategies, and issues related to recovery and remodeling. *Am J Respir Crit Care Med*(157), S. 1332-1347.

Bernard, G. R., Artigas, A., Brigham, K. L., Carlet, J., Falke, K., Hudson, L., et al. (1994). Report of the American- European Consensus Conference on Acute Respiratory Distress Syndrome: Definitions, Mechanisms, Relevant Outcomes, and Clinical Trial Coordination. *Journal of Critical Care*(9(1)), S. 72-81.

Black, S. M. (2010). New insights into acute lung injury. *Vascul Pharmacol*(52(5-6)), S. 171-174.

Matthay, M. A. (2008). Novel molecular strategy to prevent pulmonary edema. *Crit Care Med*(36(5)), S. 1671-1673.

Murray, J. F., Matthay, M. A., Luce, J. M., & Flick, M. R. (1988). An expanded definition of the adult respiratory distress syndrome. *An Rev Respir Dis*(138), S. 720-723.

Vincent, J. L., Moreno, R., Takala, J., Willatts, S., De Menddonca, A., Bruining, H., et al. (1996). The SOFA (Sepsis-related Organ Failure Assessment) score to describe organ dysfunction/failure. On behalf of the Working Group on Sepsis Related Problems of the European Society of Intensive Care Medicine. *Intensive Care Med*(22), S. 707-710.

Ware, L. B., & Matthay, M. A. (2000). The Acute Respiratory Syndrome (Review Article). *NEJM*(342(18)), S. 1334-1359.

Ware, L. B., & Matthay, M. A. (2001). Alveolar fluid clearance is impaired in the majority of patients with acute lung injury and the acute respiratory distress syndrome. *Am J Respir Crit Care Med*(163(6)), S. 1376-1383.

16. TABLES

Table 1: VISIT AND ASSESSMENT SCHEDULE 14
Table 2 Number of subjects for each possibly related adverse event 37
